# Phosvitin-Derived Peptide Pt5-1c Is a Pro-Angiogenic Agent Capable of Enhancing Wound Healing

**DOI:** 10.3390/biom16010065

**Published:** 2025-12-31

**Authors:** Cuiling Xuan, Mei Li, Peng Zhang, Yunchao Wang, Hongyan Li, Zhiqin Gao, Shicui Zhang, Fei Wu

**Affiliations:** 1 School of Life Science and Technology, Shandong Second Medical University, Weifang 261053, China; xuancuiling@stu.sdsmu.edu.cn (C.X.); limei@sdsmu.edu.cn (M.L.); 20240064@stu.sdsmu.edu.cn (P.Z.); gaozhiqin@sdsmu.edu.cn (Z.G.); 2 Institute of Evolution & Marine Biodiversity, Ocean University of China, 5 Yushan Road, Qingdao 266003, China; yunchaowang1125@stu.ouc.edu.cn (Y.W.); hongyanli@ouc.edu.cn (H.L.)

**Keywords:** antimicrobial peptide, wound healing, angiogenesis, endothelial cells, Pt5-1c

## Abstract

Antimicrobial peptides (AMPs) have been shown to have pro-angiogenic activity, capable of enhancing neovascularization and facilitating the healing of chronic wounds. However, information as such remains rather limited. Here we clearly showed that the fish phosvitin-derived AMP Pt5-1c was able to enhance angiogenesis in both murine full-thickness wound models and zebrafish with vascular defects models. We also showed that Pt5-1c was able to promote endothelial cell motility, adhesion, survival, filopodia protrusion, and induce endothelial tube formation. In addition, we found that Pt5-1c could upregulate production of proangiogenic factors including VEGF, PDGF, FGF and EGF. It was revealed that Pt5-1c promoted endothelial cell motility, growth and survival via activation both PI3K/AKT/mTOR and p38 MAPK pathways as well as HIF-1-VEGF axis. It is apparent that Pt5-1c is a novel candidate of pro-angiogenic agents for vascular regenerative therapy.

## 1. Introduction

Skin is the natural covering of the human body, consisting of a keratinized epidermis and a collagen-rich dermis, which protects humans from injuries caused by mechanical damage, extreme temperature, microbial infection, and ultraviolet radiation. These external injuries penetrate not only epidermis but also dermis, or even subcutaneous tissues causing difficulties in healing wounds [1,2]. Cutaneous wound healing is a complex and highly coordinated biological process involving four different and overlapping phases: hemostasis, inflammation, proliferation, and remodeling [3,4]. However, under pathological conditions such as diabetes, obesity, or aging, the process is frequently impaired, resulting in the formation of chronic non-healing wounds [5,6,7], which can lead to serious disturbances in patients’ quality of life and put enormous burden on the healthcare system [8,9]. A hallmark of chronic wounds is aberrant angiogenesis due to microcirculatory disorder and endothelial dysfunction [10,11,12], which leads to insufficient nutrient and oxygen supply, continuous pro-inflammatory state, impaired granulation tissue formation, and compromised re-epithelialization that collectively cause delayed wound closure or healing failure [13,14].

Angiogenesis, the formation of new blood vessels from pre-existing capillaries or post-capillary venules, is essential for effective wound repair [15,16]. It is a process that is tightly regulated by pro-angiogenic growth factors such as epidermal growth factor (EGF), fibroblast growth factor (FGF), platelet-derived growth factor (PDGF), and vascular endothelial growth factor (VEGF) [17,18,19]. These ligands bind to their respective receptors, EGF receptor (EGFR), FGF receptor (FGFR), PDGF receptor (PDGFR), and VEGF receptor (VEGFR), triggering phosphorylation and activation of downstream signaling pathways that promote cell proliferation, migration, and eventually angiogenesis [17,18]. In the case of chronic wounds, however, the expression of these factors and their receptors is significantly downregulated, thus disrupting the healing cascade. This suggests that therapy of chronic wounds may need to reactivate these signaling pathways [20].

Antimicrobial peptides (AMPs), typically ranging from 10 to 50 amino acids in length, are naturally produced by various organisms, including plants and animals, as part of the innate immune response to pathogens [21,22]. They have a broad-spectrum of antimicrobial activity against both bacteria and viruses as well as fungi and protozoa [23,24,25,26]. They also possess immunomodulatory properties [27]. In addition, many AMPs exhibit tissue-repair properties, making them promising candidates for wound therapy [22]. It has been shown that the peptide RP557 is able to enhance bacterial clearance and wound healing in diabetic mice by eliminating biofilms and strengthening innate immunity, and Andersonin-W1 (AW1) directly binds to Toll-like receptor 4 (TLR4) and modulates the TLR4/nuclear factor-κB (NF-κB) signaling axis to regulate macrophage polarization and control inflammation, thereby promoting diabetic wound healing [28,29]. Moreover, several AMPs, including AMP-IBP5, Tylotoin, CW49, Temporin A and Temporin B, have been shown to stimulate the migration and proliferation of keratinocytes and fibroblasts, accelerating re-epithelialization [1,30]. Interestingly, AMP-IBP5 can also activate Mas-related G protein-coupled receptor X2 (MrgprX2) and low-density lipoprotein receptor-related protein 1 (LRP1), increasing cell motility and survival and contribute to wound closure [30]. Besides these cellular effects, AMPs also display pro-angiogenic activity. For example, both AW1 and AMP-IBP5 are able to induce the upregulation of VEGF, α-smooth muscle actin (α-SMA), and CD31, thus enhancing neovascularization and facilitating the healing of chronic wounds [29,30].

Our previous studies have proven that Pt5-1c, a 29-amino-acid peptide derived from fish phosvitin, has both antimicrobial properties and wound healing capabilities. We have shown that Pt5-1c can function via a combined mode of action, including recognition of the microbes as a pathogen-associated molecular pattern, depolarization of bacterial membranes, and increase in membrane permeability [31]. We have also demonstrated that Pt5-1c exerts synergistic effect and antibiofilm activity with traditional antibiotics (oxacillin, vancomycin, streptomycin and azithromycin) against multidrug-resistant (MDR) bacteria growing as biofilms in vitro and in vivo [32]. Very recently, we found that Pt5-1c is able to promote re-epithelialization and granulation tissue formation in a murine full-thickness wound model via activation of the EGFR pathway, and enhance the adhesion, migration, and proliferation of fibroblasts and keratinocytes [33,34]. In addition, Pt5-1c can stimulate the synthesis of α-SMA, induce fibroblast differentiation, and facilitate collagen gel contraction [33]. However, it remains open if Pt5-1c has any pro-angiogenic property. The present study is thus conducted to answer the question using mice, transgenic zebrafish and endothelial cell lines as models in vivo and in vitro, respectively.

## 2. Materials and Methods

### 2.1. Antimicrobial Peptide Pt5-1c

Fish phosvitin-derived AMP Pt5-1c was synthesized by Sangon Biological Engineering Technology & Services Co., Ltd. (Shanghai, China), using standard solid phase 9-fluorenylmethoxy carbonyl (FMOC) method [33]. Pt5-1c synthesized was purified >95% by reverse-phase high-performance liquid chromatography (HPLC) and verified by mass spectrometer. The final products were lyophilized and stored at −80 °C until use. The amino acid sequence of Pt5-1c is SRMKKWAKIIEKWRKWHKKRWLAHHSATK and its observed molecular weight is 3755 Da.

### 2.2. Cell Culture

Human umbilical vein endothelial cells (HUVECs), provided by Dr. Hongyan Li (Institute of Evolution and Marine Biodiversity, Ocean University of China), were cultured in RPMI 1640 medium (Gibco, Grand Island, NY, USA) supplemented with 10% fetal bovine serum (FBS) (Gibco, Grand Island, NY, USA) and 1% penicillin-streptomycin solution (Cytiva, Marlborough, MA, USA) [35]. The cells were kept at 37 °C in a humidified cell incubator containing 5% CO_2_. The medium was replaced every other day and passaged when the cells attained ~80% confluence. Both human embryonic fibroblasts (HELF) cells and human epidermal keratinocytes (HaCaT) cells were cultured as previously described [33,34].

### 2.3. Scratch Wound Healing Assay

The migration ability of HUVECs induced by Pt5-1c was tested by scratch wound healing assay. Briefly, HUVECs (5 × 10^5^ cells per well) were seeded into 6-well microplate, and grown to reach confluence at 37 °C. After starvation for 12 h in the medium without FBS, the cell monolayer was scratched with 200 μL pipette tip and washed with PBS to remove cellular debris. For inhibitor studies, HUVECs were pre-incubated with 10 μM wortmannin (an inhibitor of PI3K, Beyotime, Shanghai, China) or 10 μM SB203580 (an inhibitor of p38 MAPK, Beyotime, Shanghai, China) for 2 h prior to scratching. Subsequently, the serum-free medium with or without Pt5-1c was added to each well, and the photomicrographs of the wounds were captured at 0, 12, and 24 h by the microscope (Leica DMI3000 B, Weztlar, Germany). The wound areas were quantified using ImageJ software (Version 1.52, National Institutes of Health, Bethesda, MD, USA). The scale was set for each image based on the embedded micrometer. The wound margins were then manually traced using the Polygon selection tool, and the area was measured. Scratch healing rate (%) = (C_0_ − C_t_)/C_0_ × 100%, where C_0_ represents the scratch area at 0 h and C_t_ represents the scratch area at specific times.

### 2.4. Transwell Assay

Transwell assay was conducted to specifically quantify directed cell migration (chemotaxis) in response to a chemoattractant gradient in vitro [36]. Briefly, HUVECs (3 × 10^4^ cells/well) were starved for 24 h in medium with 2% FBS, resuspended in the medium containing 2% FBS with or without Pt5-1c, and placed on the upper chamber of a transwell cell culture insert. An aliquot of 600 μL of the medium containing 20% FBS was added to the lower chamber of the transwell cell culture insert. After incubation for 24 h, the cells that had migrated through the membrane were fixed with 4% paraformaldehyde (PFA) (Solarbio, Beijing, China) for 20 min, and were stained with 0.1% crystal violet (Solarbio, Beijing, China) for 20 min. Cell images were captured by the microscope (Olympus BX53E2C, Tokyo, Japan), and ImageJ software (NIH, Bethesda, MD, USA) was used to quantify the number of migrated cells on the lower surface of the member.

### 2.5. F-Actin Staining

To assess cell morphology, HUVECs (5 × 10^4^ cells per well) were seeded into 24-well microplate and grown to reach confluence at 37 °C. After starvation for 12 h in medium without FBS, the serum-free medium with or without Pt5-1c was added to each well. After culture for 24 h, HUVECs were fixed with 4% PFA for 20 min, permeabilized with 0.5% Triton X-100 (Solarbio, Beijing, China) for 15 min, and blocked with 5% bovine serum albumin (BSA; Sigma, St. Louis, MO, USA) at room temperature for 30 min. The F-actin fibers were stained using rhodamine (TRITC)-labeled phalloidin (Yeasen, Shanghai, China), and the nuclei of cells counterstained with Hoechst 33342 for 10 min according to the manufacturer’s instructions. The cells were observed and photographed under the confocal microscope (Leica SP8, Wetzlar, Germany).

### 2.6. Cell Adhesion Assay

In total, 12-well microplates were coated with fibronectin (2 μg/mL) (Sigma, St. Louis, MO, USA), and placed at 37 °C for 2 h. HUVECs were starved for 12 h in medium without FBS, and then collected, resuspended in the serum-free medium with or without Pt5-1c, and allowed to attach onto the fibronectin-coated microplates for 15, 30, 60, and 120 min, respectively. The cells were fixed in 4% PFA, and the cell adhesion was then detected by crystal violet staining for 30 min. At least 10 fields from each sample were observed, photographed, and used to count the adherent cells under the microscope (Leica DMI3000 B, Wetzlar, Germany).

### 2.7. Cell Proliferation Assay

First, HUVEC cell proliferation was evaluated by immunocytochemical detection of cells that permitted incorporation of 5-ethynyl-2′-deoxyuridine (EdU) into cellular DNA using Yeflour 594 EdU Imaging kit (Yeasen, Shanghai, China), according to the manufacturer’s instructions. Briefly, after starvation for 12 h in medium without FBS, HUVECs (5 × 10^4^ cells per well) were seeded into 24-well microplate, treated with or without Pt5-1c for 24 h and then incubated with 10 μM EdU for 2 h. The cells were fixed with 4% PFA for 30 min, and followed by neutralization with glycine solution (2 mg/mL) for 5 min. The fixed cells were permeabilized with 0.5% Triton X-100 in PBS for 20 min and then incubated with a Click-iT reaction mixture for 30 min under dark. The nuclear DNA was counterstained using DAPI, and the EdU-positive images were observed and recorded under confocal microscopy (Leica SP8, Wetzlar, Germany). Second, HUVEC cell proliferation was measured by the cell growth assays [37]. After starvation for 12 h in medium without FBS, HUVECs (1 × 10^5^ cells per well) were seeded into 12-well microplate overnight at 37 °C. The cells were then incubated with Pt5-1c in the serum-free medium in duplicate for 24 and 48 h, respectively. The cells were then gently washed with PBS to remove most detached, non-viable cells, and the adherent cells were detached from the culture plates using a trypsin-EDTA (0.25%) solution (Cytiva, Marlborough, MA, USA), and the number of live cells was counted. Thirdly, HUVEC cell proliferation was determined by WST-8 hydrolysis using Cell Counting Kit-8 (CCK-8, Beyotime, Shanghai, China) assays. HUVECs (8 × 10^3^ cells per well) were seeded into 96-well microplate and placed at 37 °C overnight. The cells were incubated with Pt5-1c in the serum-free medium in duplicate for 24 and 48 h, respectively, and an aliquot of 10 μL of WST-8 solution was then added to each well, followed by incubation for an additional 1 h. The optical density (OD) was detected at 450 nm by the microplate reader (Tecan Spark, Shanghai, China).

### 2.8. Assay for Tube Formation In Vitro

Matrigel Matrix Basement Membrane (Corning, Corning, NY, USA) was added to 96-well microplate (50 μL/well), and incubated at 37 °C to gel for 30 min. After starvation for 12 h in medium without FBS, HUVECs were collected and seeded into matrigel-coated 96-well microplate (5 × 10^4^ cells/well) in the serum-free medium with or without Pt5-1c. After incubation for 4 h at 37 °C, tube formation was detected under the microscope (Leica DMI3000 B, Wetzlar, Germany). For inhibitor study, HUVECs were pre-incubated with 10 μM wortmannin or 10 μM SB203580 for 2 h prior to seeding. The total length and the number of nodes showing the activity of tube formation of HUVECs were monitored by ImageJ software (NIH, Bethesda, MD, USA).

### 2.9. Visualization of Zebrafish Vascular System

The transgenic (Tg) zebrafish line, Tg (*flk: mCherry*), expressing enhanced red fluorescent protein in endothelial cells, enables direct visualization of the vascular system [35]. The Tg (*flk: mCherry*) zebrafish was provided by Dr. Hongyan Li (Institute of Evolution and Marine Biodiversity, Ocean University of China), maintained at 27 ± 1 °C under a 14 h for light and 10 h for dark cycle, and fed twice a day. The vatalanib PTK787, an inhibitor of VEGFR, was purchased from Beyotime (Shanghai, China), and used to induce intersegmental vascular deficiency model according to the method previously reported [17]. The Tg embryos were produced by natural pairwise mating and raised at 28 °C in E3 medium. The healthy hatched larvae were picked up at 24 h post-fertilization (hpf), immersed in saline only (control group), or in saline with 0.2 μg/mL PTK787 (model group), 0.2 μg/mL PTK787 plus 0.5 μg/mL Pt5-1c (Experimental group 1), or 0.2 μg/mL PTK787 plus 1.0 μg/mL Pt5-1c (experimental group 2), and distributed into 12-well microplate (15 larvae/well). After pre-incubation for 3 h, the larvae in the model group, experimental group 1 and experimental group 2 were divided into two sub-groups, and cultured in the medium with or without Pt5-1c at 28 °C. The larvae in the control group were similarly cultured with the medium. After culture for 24 h, the intersegmental vessels (ISVs) of the larvae in each group were detected under confocal microscopy (Leica SP8, Wetzlar, Germany), and the ISV lengths quantified using the ImageJ software (NIH, Bethesda, MD, USA).

### 2.10. Establishment of Mice Dorsal Skin Wound Model

All the animal experiments of this study were carried out in accordance with the guiding principles by the Ethics Committee of the Laboratory Animal Administration of Shandong Second Medical University in Shandong Province (For mice, Approval Code: 2024SDL521, Approval Date: 7 June 2024; For rats, Approval Code: 2024SDL231, Approval Date: 7 March 2024).

A total of forty male ICR mice, aged 6–8 weeks old, were purchased from Pengyue Laboratory Animal Technology (Shandong, China) (ID: SCXK 20190003), housed in specific pathogen-free conditions with a controlled temperature (25 ± 3 °C), humidity (55 ± 10%) and a 12 h light/dark cycle (lighting: 8:00–20:00), and fed with a purified diet and water. The dorsal skin wound model was established as previously described [33]. Briefly, the mice were anesthetized with 2% sodium pentobarbital (Solarbio, Beijing, China), and their dorsal hairs removed with an electric clipper and depilatory cream. The skins were then cleaned with PBS and the full-thickness wound was built on the dorsal region using a biopsy punch with 8 mm-diameter. The mice were then divided into two groups at random (*n* = 20): the control group and experimental group. The mice of the control group were treated with 20 μL of sterile saline per wound site twice daily for 12 days, and those of the experimental group treated with 20 μL of 10 μg/mL Pt5-1c similarly. On days 3, 6, 9, 10, 12 post-wounding, three mice were randomly sampled from each group at each time point, and sacrificed, and the tissue involving the wound was obtained from the mice and harvested. Part of each tissue specimen was fixed in 4% PFA for immunohistochemical study, and the remaining part of the tissue was frozen in liquid nitrogen till used.

### 2.11. Immunofluorescence and Immunohistochemical Analysis

To detect angiogenesis in the wound after intervention, the immunohistochemical staining was performed using CD31, α-SMA, and VEGFA antibodies as previously described [38]. The fixed tissue specimens were dehydrated, embedded in paraffin, and sectioned at a thickness of 5 μm. The sections were dewaxed, rehydrated, and treated with 3% hydrogen peroxide for 15 min. After placing in a citric acid buffer for 10 min for antigen retrieval, the sections were incubated with goat serum albumin (Solarbio, Beijing, China) for 30 min. Subsequently, they were incubated with the primary antibodies including rat anti-CD31 (1:200; Santa Cruz, Dallas, TX , USA), rabbit anti-α-SMA (1:200; ABmart, Shanghai, China) and rabbit anti-VEGFA (1:100; Proteintech, Rosemont, IL, USA) overnight at 4 °C, and then with the Alexa Fluor^TM^546-conjugated goat anti-rat secondary antibody (1:400; Invitrogen, Waltham, MA, USA), FITC-labeled goat anti-rabbit secondary antibody (1:400; Beyotime, Shanghai, China), and HRP-conjugated goat anti-rabbit secondary antibody (1:50; ImmunoWay, Plano, TX, USA) for 60 min at room temperature. Finally, for immunohistochemical analysis, the sections were stained with a DAB kit (Beyotime, Shanghai, China), and the nuclei were counterstained with hematoxylin (Solarbio, Beijing, China); for immunofluorescence staining, the nuclei were stained with DAPI (Solarbio, Beijing, China). All the sections were observed, and photographed with a microscope (Olympus BX53E2C, Tokyo, Japan). For quantitative analysis, we randomly selected 3–5 fields in each tissue section based on the size of the granulation tissue using ImageJ software (NIH, Bethesda, MD, USA).

### 2.12. Assessment of Pt5-1c Toxicity

The assessment of Pt5-1c toxicity was conducted as previously described [39]. The 8-week-old male Wistar rats purchased from Pengyue Laboratory Animal Technology (ID: SCXK 20220006) were exploited to make a dorsal skin wound model and treated with Pt5-1c above (Section 2.10). Three rats were taken from each group, and treated with normal saline (Negative control), Pt5-1c (Experimental group), or VEGFA (Positive control). Twelve days later, the rats were euthanized with sodium pentobarbital (120 mg/kg), and the tissues including heart, liver, spleen, lung, and kidney were dissected out and used for H&E (Solarbio, Beijing, China) staining. The cell morphology and tissue structure were observed and photographed under the microscope (Olympus BX53E2C, Tokyo, Japan).

### 2.13. Western Blot Assay

HUVECs were seeded in a 60 mm culture dish and cultured at 37 °C. After reaching 60% confluence, the cells were starved overnight in medium without FBS and then treated with 1 μg/mL of Pt5-1c for 5, 15, 30, and 60 min, respectively. The cells were then detached from the culture dish and lysed using the lysis buffer (20 mM Tris pH7.5, 150 mM NaCl, 1% Triton X-100) containing protease and phosphatase inhibitor cocktail (1×) (Beyotime, Shanghai, China) at 4 °C for 30 min. The lysates were centrifugated at 12,000× *g* at 4 °C for 15 min, and the supernatants were pooled. For the wound skin tissues harvested above, they were homogenized in 1× PBS (pH 7.4) containing protease and phosphatase inhibitor cocktail using Polytron and sonicator. The homogenates were centrifuged at 4 °C at 12,000× *g* for 15 min, and the supernatants were pooled. Protein concentration was detected using a BCA Protein Assay Kit (Beyotime, Shanghai, China). Equal amounts of total protein (40 μg) were electrophoresed on 10–15% sodium dodecyl sulfate-polyacrylamide gel electrophoresis and then transferred to polyvinylidene fluoride (PVDF) membranes (Millipore, Burlington, MA, USA). The membranes were blocked with 5% BSA in PBST for 2 h at room temperature, followed by incubation overnight at 4 °C with the primary antibodies against VEGFA (1:1000; Proteintech, Rosemont, IL, USA), AKT (1:1000; Proteintech), phospho-AKT (1:1000; Proteintech), phospho-mTOR (1:1000; Proteintech), mTOR (1:1000; Proteintech), p38 (1:1000, ABclonal, Wuhan, China), and phospho-p38 (1:1000, Cell Signaling Technology, Beverly, MA, USA), respectively. After washing three times with PBST, the membranes were incubated with corresponding HRP-conjugated secondary antibodies (1:5000; Proteintech) at room temperature for 1 h. The membranes were then incubated with ECL kit (Epizyme, Shanghai, China) and observed using Mini Chemifluorescence imaging analysis system. The band intensity was analyzed using ImageJ software (NIH, Bethesda, MD, USA).

### 2.14. Quantitative Real-Time Polymerase Chain Reaction (qRT-PCR)

HUVECs, HaCaT cells, and HELF cells were seeded into 6-well microplate, and treated with or without Pt5-1c (1 μg/mL) for 24 h. RNAs were extracted from cells, and wound skin tissues of the mice harvested above with RNAiso plus (TaKaRa, Kyoto, Japan). The cDNAs were synthesized with Prime-Script™ RT reagent Kit with gDNA Eraser (TaKaRa) according to the manufacturer’s instructions and stored at −20 °C until use. The gene expression of pro-angiogenic growth factors was examined by qRT-PCR as previously described [40]. Primer Premier 5.0 program was used to design the specific primers of each gene. The human-*GAPDH* gene or mice-*Gapdh* gene were chosen as for internal standardization, respectively. The relative expression levels of each gene were assessed using the 2^−^^ΔΔCT^ method. The primer sequences used for human cells and mice tissues were listed in Table 1 and Table 2.

### 2.15. Molecular Docking

The corresponding protein structure of PI3K (PDB ID:4UWH), AKT (PDB ID:8UW9), mTOR (PDB ID:1AUE), and p38 (PDB ID:1WBV) was obtained from RCSB Protein Data Bank (https://www.rcsb.org/ (accessed on 11 September 2025)). Molecular docking was performed using the HDOCK server (http://hdock.phys.hust.edu.cn/ (accessed on 22 September 2025)) to predict the preferential binding site of Pt5-1c on the target protein. The docking poses were visualized with PyMOL （Version 1.7, Schrödinger, LLC, New York, NY, USA） tools. The binding energy was calculated using the PDBePISA website (https://www.ebi.ac.uk/pdbe/pisa/ (accessed on 26 September 2025)).

### 2.16. Statistical Analysis

The experimental data were expressed as mean ± SD with GraphPad Prism Software (Version 9.0, GraphPad Software, Inc., San Diego, CA, USA). Statistical Significance was determined using Student’s *t*-test or one-way ANOVA. *p* values of less than 0.05 were considered significant differences (* *p* < 0.05, ** *p* < 0.01 and *** *p* < 0.001 indicated statistically significant compared with the control group. # *p* < 0.05, ## *p* < 0.01 and ### *p* < 0.001 indicated as statistically significant compared with the model group).

## 3. Results

### 3.1. Pt5-1c Promoted Motility, Adhesion, Survival, and Filopodia Protrusion of Endothelial Cell

**Figure 1:**
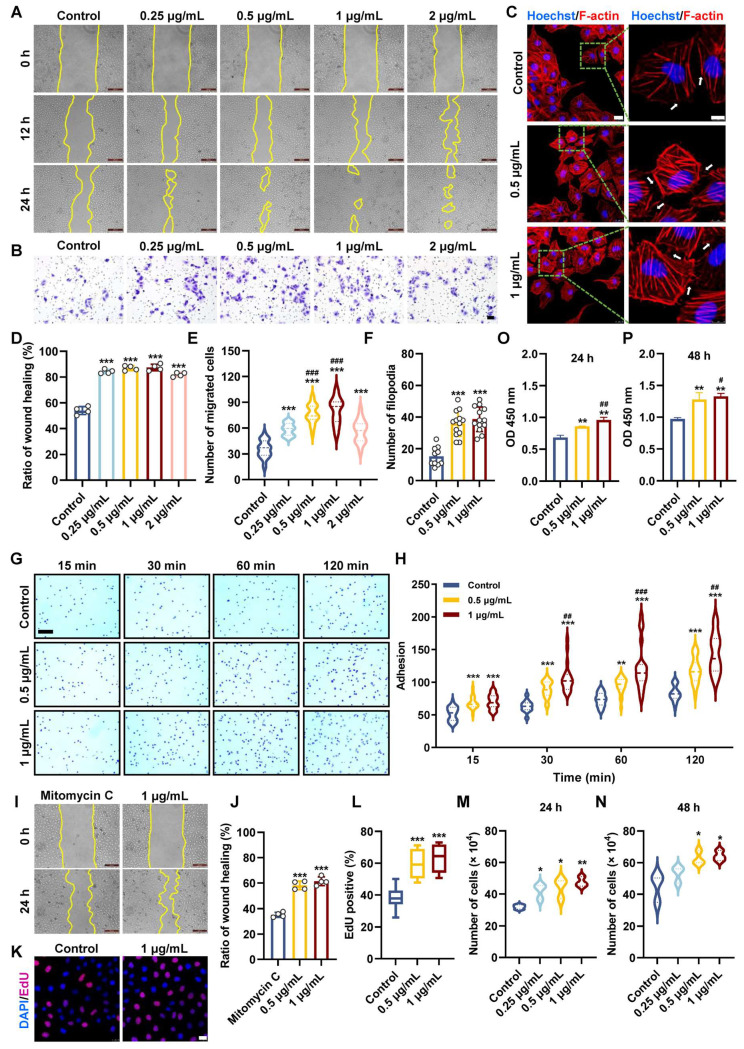
Pt5-1c promoted motility, adhesion, survival, and filopodia protrusion of endothelial cell. (**A**,**D**) HUVECs wounds were created and then treated with Pt5–1c at 0.25–2 μg/mL for 12 h and 24 h. Representative images in HUVECs after treatment with Pt5-1c. Wound healing was evaluated by measuring the area between the wound margins. Scale bar: 200 μm; (**B**,**E**) Transwell assay examined the migratory abilities of HUVEC after exposure to Pt5-1c for 24 h. Quantification of the corresponding migration cells (*n* = 3). Scale bar = 200 μm; (**C**,**F**) Representative images of F-actin staining of HUVECs treated with Pt5-1c and quantitative analysis of filopodia. Inset represents the magnification of the image portion indicated by the green box. White arrows indicate filopodia formation. The scale bars are 25 μm and 10 μm, respectively. Hoechst 33342 is used as nuclear stain. Data are shown as means ± SD (*n* = 10 microscope fields); (**G**,**H**) Representative images and statistical analysis of cell adhesion. Scale bar: 200 μm; (**I**,**J**) Representative images show that Pt5-1c reversed the mitomycin C-induced inhibition of cell proliferation and increased the wound healing rate. Wound healing was evaluated by measuring the area between the wound margins. Scale bar: 200 μm; (**K**,**L**) The effect of Pt5-1c on endothelial cell proliferation was detected by EdU incorporation assay and quantitatively analyzed. In every group, 10 microscope fields were randomly captured. Scale bar: 25 μm; (**M**,**N**) The number of HUVECs treated with different concentration of Pt5-1c for 24 h or 48 h; (**O**,**P**) Cell viability was determined using CCK-8 with the OD measured at 450 nm using a microplate reader. Data are shown as means ± SD (*n* = 3). * *p* < 0.05, ** *p* < 0.01, *** *p* < 0.001, compared with control group; # *p* < 0.05, ## *p* < 0.01, ### *p* < 0.001, compared with Pt5-1c (0.5 μg/mL)-treated group.

Endothelial cell migration, involving membrane elongation to protrude filopodia, is fundamental to the process of angiogenesis [41]. To evaluate if Pt5-1c could promote the migration of endothelial cells, both scratch and transwell assays were performed. As illustrated in Figure 1A,D, Pt5-1c was able to increase the scratch wound healing in a time- and dose-dependent fashion. Compared with control, HUVECs incubated with Pt5-1c showed a significant increase in the wound healing (*p* < 0.001), with the highest healing rate of 87.4% at 1 μg/mL of Pt5-1c at 24 h, that was 61.9% higher than those of control group. Similarly, transwell assay revealed that Pt5-1c displayed a significant invasive ability across the membrane in the concentration range of 0.25 μg/mL to 2 μg/mL, with the highest invasive ability at 1 μg/mL of Pt5-1c (Figure 1B,E). Therefore, 0.25, 0.5, and 1 μg/mL were selected as the optimal concentrations in subsequent experiments. These manifested that Pt5-1c can promote the migration of endothelial cells, consistent with the previous reports for fibroblasts [33] and keratinocytes [34].

The effect of Pt5-1c on filopodia formation was examined using phalloidin staining to label F-actin. It showed that a considerable density of filopodia was observed in HUVECs incubated with Pt5-1c in comparison with control (Figure 1C,F). As cell migration depends on cell-extracellular matrix adhesiveness, the effect of Pt5-1c on the adhesion of HUVECs to fibronectin, a major substrate for endothelial cell [42], was also determined. As revealed by Figure 1G,H, Pt5-1c could significantly increase the adhesion of HUVECs to fibronectin in a time- and dose-dependent manner.

In addition to migration, proliferation of endothelial cells is also crucial for normal angiogenesis [43]. We first tested the effect of Pt5-1c on the proliferation of HUVEC cell treated with mitomycin C, a cell division inhibitor. As shown in Figure 1I,J, Pt5-1c was able to reverse the inhibitory effect of mitomycin C on cell proliferation and enhance the wound healing rate. We then examined the effect of Pt5-1c on the proliferative ability of HUVECs incubated with EdU. Treatment with Pt5-1c resulted in a significant increase in the proportion of EdU-positive HUVECs (*p* < 0.001, Figure 1K,L). Specifically, the percentage of EdU-positive HUVECs in the control group was 38.4%, while the percentage of EdU-positive HUVECs in the 0.5 μg/mL and 1 μg/mL Pt5-1c-treated groups was 60% and 63.5%, respectively. Similarly, treatment with Pt5-1c also resulted in a marked rise in total cell number (*p* < 0.05, Figure 1M,N). At 24 and 48 h after treatment with 1 μg/mL Pt5-1c, the total cell numbers were about 47.6 × 10^4^ and 64.2 × 10^4^ cells, respectively, while the total cell numbers in the control group were 31.9 × 10^4^ and 45.2 × 10^4^ cells at the same time points. Consistent with the above observations, the CCK-8 assay also confirmed that treatment with Pt5-1c significantly enhanced cell viability and proliferation of endothelial cells (Figure 1O,P). After 48 h treatment, the OD was 1.33 in cells treated with 1 μg/mL of Pt5-1c, compared to 0.97 in the control group (*p* < 0.05, Figure 1O,P). All the data aforementioned indicate that Pt5-1c can promote the motility, adhesion, survival, and filopodia protrusion of endothelial cells.

### 3.2. Pt5-1c Promoted Both Tube Formation and Proangiogenic Factor Production

**Figure 2:**
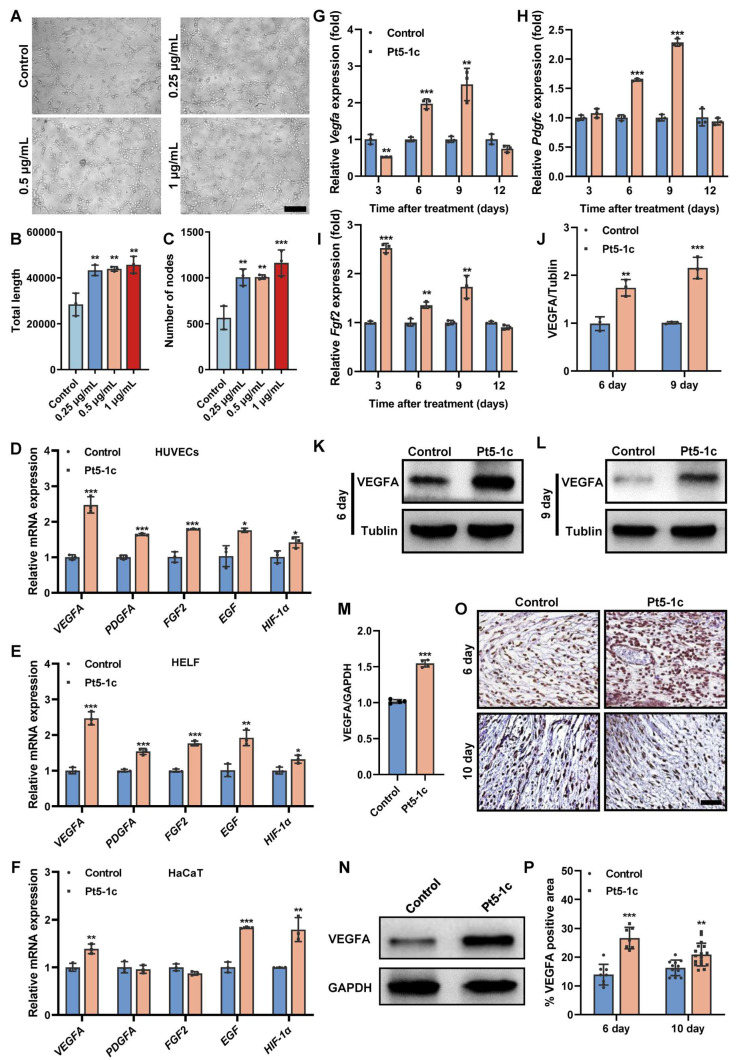
Pt5-1c promoted both tube formation and proangiogenic factor production. (**A**–**C**) The angiogenesis effect of Pt5-1c was detected by in vitro tube formation assay. Representative images of tube-like structures (**A**), and quantification of the total tube length (**B**) and the number of nodes (**C**). Scale bar: 200 μm. Data are shown as means ± SD *(n* = 3); (**D**–**F**) qRT-PCR analysis of the gene expression of *VEGFA*, *PDGFA*, *FGF2*, *EGF* and *HIF-1α* in HUVECs, HELF and HaCaT cells, respectively, after treatment with Pt5-1c (1 μg/mL) for 24 h. Data are shown as means ± SD (*n* = 3); (**G**–**I**) qRT-PCR analysis of the gene expression of *Vegfa*, *Pdgfc* and *Fgf2* in the wound tissues receiving different treatment on days 3, 6, 9 and 12 post-wounding (*n* = 3); (**J**–**L**) Western blot analysis of the VEGFA protein expression in skin wound tissues of mice treated with Pt5-1c (10 μg/mL) or sterile saline (control) at 6 d and 9 d post-wounding (*n* = 3); (**M**,**N**) VEGFA protein expression was assessed by Western blot after Pt5-1c treatment for 24 h in HUVECs (*n* = 3); (**O**,**P**) Representative images of immunohistochemical staining for the VEGFA and quantitative analysis the area of VEGFA-positive cells in skin wound sites of mice treated with Pt5-1c (10 μg/mL) or sterile saline (control) at 6 d and 10 d post-wounding. Scale bar: 100 μm. Statistical differences between control and Pt5-1c-treated samples are assessed using unpaired *t*-test, * *p* < 0.05, ** *p* < 0.01, *** *p* < 0.001. Original images can be found in Supplementary Material.

Tube formation assay revealed that compared with control, Pt5-1c caused a marked increase in the total capillary length and number of nodes (*p* < 0.01, Figure 2A–C), suggesting it has the capacity to induce tube formation in endothelial cells. Therefore, we next examined the effect of Pt5-1c on the expression of key angiogenic growth factors in HUVECs, HaCaT, and HELF cells at a concentration of 1 μg/mL, which was found to be optimal for its pro-angiogenic activity [44]. It was found that treatment with Pt5-1c upregulated the expression of *VEGFA*, *PDGFA*, *FGF2*, *EGF*, and *HIF-1α* (hypoxia-inducible factor-1α) in both HUVECs and HELF cells (Figure 2D,E). Similarly, Pt5-1c was also shown to induce an increase in the expression of *VEGFA*, *EGF*, and *HIF-1α* in HaCaT cells, though little difference was observed in the expression of *PDGFA* or *FGF2* in comparison with control (Figure 2F). In agreement with the results above, Pt5-1c administration also significantly enhanced the expression of *Vegfa* (*p* < 0.01; Figure 2G), *Pdgfc* (*p* < 0.001; Figure 2H), and *Fgf2* (*p* < 0.01; Figure 2I) in a murine full-thickness wound model. Moreover, both Western blot and immunohistochemical analysis demonstrated that Pt5-1c treatment significantly increased the synthesis of VEGFA protein in vitro (Figure 2M,N) and in vivo (Figure 2J–L,O,P). Collectively, these suggest that Pt5-1c is able to promote the tube formation via upregulating the expression of critical pro-angiogenic growth factors.

### 3.3. Pt5-1c Promoted Angiogenesis in Zebrafish with Vascular Defects

**Figure 3:**
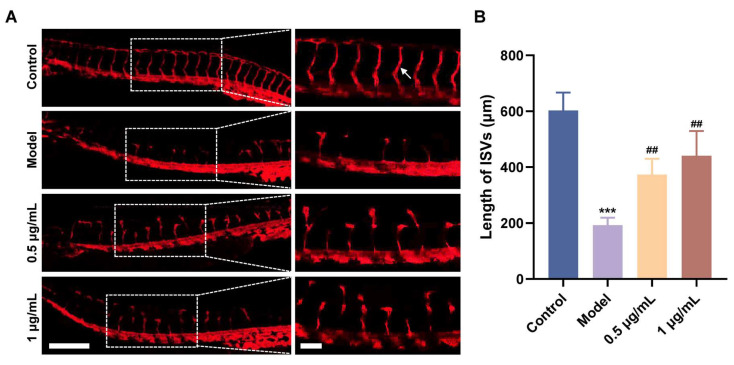
Pt5-1c promoted angiogenesis in zebrafish with vascular defects. (**A**) Fluorescence images of different groups in Tg (*flk: mCherry*) zebrafish. Insets represent the magnification of the image portion indicated by the white dotted box. The scale bars are 250 μm and 75 μm, respectively. White arrow indicates a typical ISV; (**B**) Statistical analysis of the total length of ISVs. *** *p* < 0.001 vs. control group, ## *p* < 0.01 vs. PTK787 group.

The Tg (*flk: mCherry*) zebrafish line was used to establish the vascular deficiency model by PTK787, which has been shown to impair angiogenesis by blocking the autophosphorylation of VEGFR and inhibiting endothelial cell proliferation [17]. The ISVs were detected and photographed under laser confocal microscopy. It was observed that compared with control, angiogenesis of ISVs in the model zebrafish was considerably inhibited by treatment with PTK787, and the length of ISVs was decreased by 68% (Figure 3A,B). By contrast, administration of Pt5-1c could reverse the PTK787-induced vascular defects of zebrafish in a dose-dependent manner and remarkably restored the angiogenesis and length of ISVs (*p* < 0.01), with a recovery rate of more than 60% at 1 μg/mL of Pt5-1c (Figure 3A,B). These showed that Pt5-1c can restore the formation and growth of ISVs in zebrafish with vascular defects, further confirming the presence of pro-angiogenic activity of Pt5-1c.

### 3.4. Pt5-1c Enhanced Angiogenesis in Wound Sites

**Figure 4:**
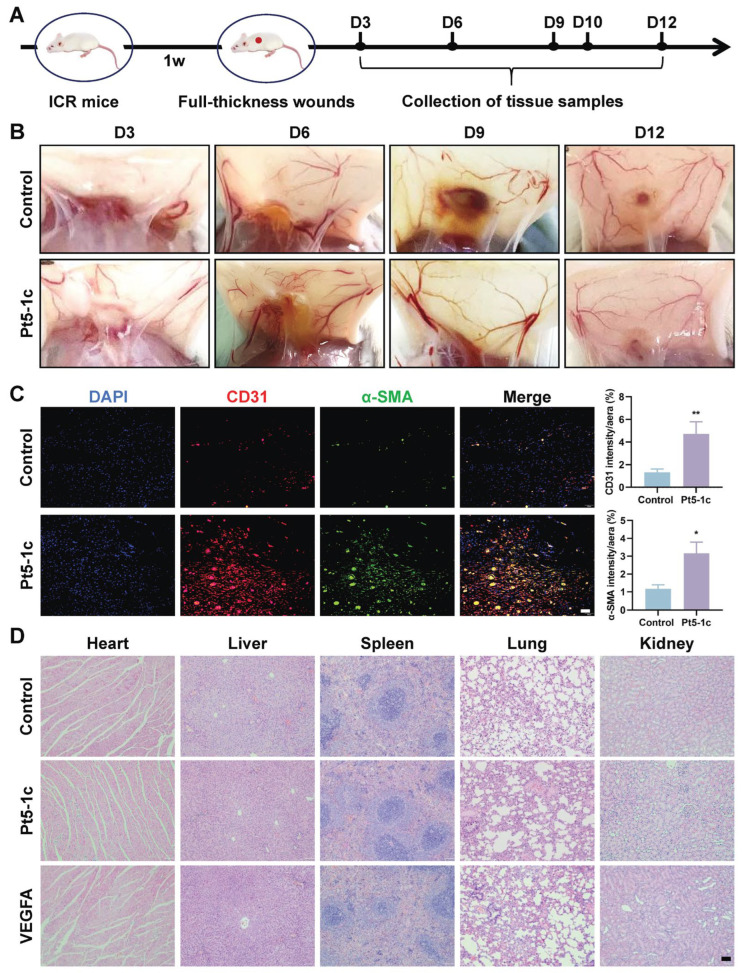
Pt5-1c enhanced angiogenesis in wound sites. (**A**) Schematic diagram of wound healing in mice using antimicrobial peptide Pt5-1c; (**B**) Representative images of blood vessels of the wounds at days 3, 6, 9, and 12 post wounding in the mice treated with Pt5-1c (10 μg/mL) or sterile saline (control); (**C**) Representation and quantification of immunostaining for CD31 or α-SMA-positive vessels at day 9 post-wounding in the mice treated with Pt5-1c (10 μg/mL) or sterile saline (control). Scale bar: 50 μm; (**D**) H&E staining images of the heart, liver, spleen, lung, and kidneys of rats after treatment with Pt5-1c for 12 days. Scale bar: 100 μm. Data are shown as means ± SEM (*n* = 3), * *p* < 0.05, ** *p* < 0.01, compared with control group.

Our previous study proved that treatment with Pt5-1c accelerated postoperative wound healing and promoted the formation of well-structured granulation tissue in the dermis, characterized by proliferation of fibroblasts and keratinocytes [33,34]. Further analysis of the capillary networks in the subcutaneous wound area demonstrated that the number of nodes and total vessel length in Pt5-1c-treated mice were markedly greater than those of the control group on post-injury days 6 and 9 (Figure 4B), suggesting that Pt5-1c has an effect of promoting wound vascularization. Next, we performed immunofluorescence staining for CD31 (an endothelial cell marker) and α-SMA (a vascular smooth muscle cell marker) to observe the vascular patterns induced by Pt5-1c on day 9 post-injury (Figure 4C). CD31 immunofluorescence staining revealed a remarkably dense population of red CD31-positive cells (red fluorescence) in the Pt5-1c group compared to that in the control group, indicating that dense microvascular structures were formed in the wounds of the Pt5-1c group. Moreover, the green fluorescence of α-SMA, which represents neonatally mature blood vessels, is more pronounced in the Pt5-1c group, confirming that Pt5-1c was able to promote angiogenesis in wound repair. Pt5-1c has been previously shown to have no hemolytic activity in vitro [31]. Here we further evaluated its biosafety under conditions in vivo. As revealed by histopathological examination of H&E-stained sections, none of the tissues including heart, liver, spleen, lung, and kidney of treated rats showed any signs of damage, fibrosis, or other morphological abnormalities following Pt5-1c administration (Figure 4D), suggesting its safety in vivo. All the data above denoted that Pt5-1c has a capacity to enhance angiogenesis and promote wound repair in a full-thickness wound model, while exhibiting a favorable biocompatibility.

### 3.5. Pt5-1c Enhanced Angiogenesis via Activating PI3K/AKT/mTOR and p38 MAPK Pathways

**Figure 5:**
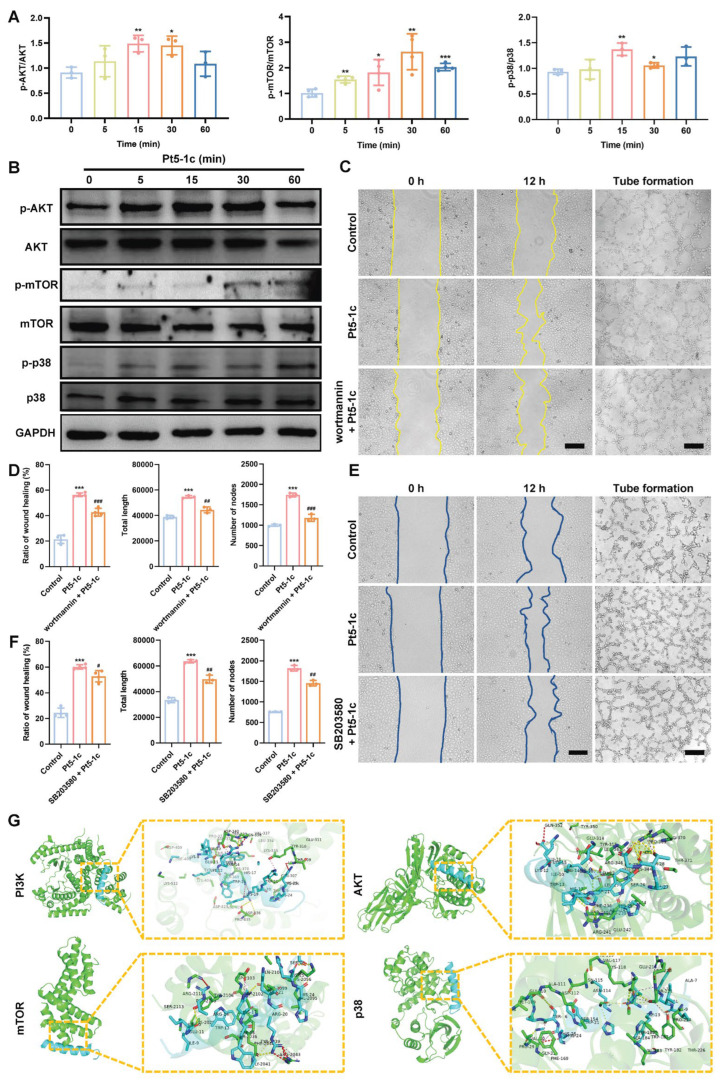
Pt5-1c enhanced angiogenesis via activating PI3K/AKT/mTOR and p38 MAPK pathways. (**A**,**B**) Western blot analysis of the phosphorylation level of AKT, mTOR, and p38 in HUVECs treated with 1 μg/mL of Pt5-1c for the indicated of time; (**C**–**F**) Cell wound healing and tube formation were measured in HUVECs that were treated with Pt5-1c (1 μg/mL) after pre-incubation with 10 μM wortmannin or 10 μM SB230580 for 2 h. Representative photomicrographs and quantitative analysis of ratio of wound healing, the total length and the number of nodes were shown. Scale bar: 200 μm. Data are expressed as means ± SD (*n* = 3); (**G**) Molecular docking pattern of Pt5-1c and target kinases presented in three dimensions (3D). The cyan is antimicrobial peptide Pt5-1c, green is target kinases presented. The rod-shaped structures represent the docking interface residues: the cyan rod-shaped structures are the Pt5-1c residues, and the green rod-shaped structures are the target kinases residues. The black font marks the key linking amino acid residues of the interface Pt5-1c to the target kinases, and the yellow and purple dotted line represents the hydrogen bond. * *p* < 0.05, ** *p* < 0.01, *** *p* < 0.001 vs. control group; # *p* < 0.05, ## *p* < 0.01, ### *p* < 0.001 vs. Pt5-1c group. Original images can be found in Supplementary Material.

Multiple signaling pathways, in particular PI3K/AKT and p38 MAPK, have been shown to promote endothelial cell functions that are essential for angiogenesis. As shown in Figure 5A,B, Pt5-1c administration caused a marked increase in the phosphorylation of AKT, mTOR, and p38 was observed in HUVECs, while little change was observed in the total protein levels of these kinases. Moreover, the blockades of the PI3K/AKT/mTOR and p38 MAPK signaling pathways by pre-incubation with wortmannin (a PI3K inhibitor) and SB230580 (a p38 MAPK inhibitor) obviously impaired the ability of Pt5-1c to promote the migration and angiogenesis of endothelial cells, as evidenced by reduced wound closure, and decreased total capillary length and node number of HUVECs (Figure 5C–F). In addition, we also assessed the binding affinity of Pt5-1c with the kinases by conducting molecular docking analysis. The estimations of binding geometric 3D structures between Pt5-1c and targets were given in Figure 5G, and the calculated binding energy of PI3K, AKT, mTOR, and p38 with Pt5-1c was calculated as −4.1, −5.0, −8.1, and −8.7 kcal/mol, respectively, suggesting that Pt5-1c demonstrated favorable binding affinity to all four targets. These revealed that the promotion of angiogenesis by Pt5-1c involves modulation of the PI3K/AKT/mTOR and p38 MAPK signaling pathways.

## 4. Discussion

Normal wound healing is a tightly regulated “angiogenic privilege phase”, which enables a transient 2–3-fold increase in local vascular density, essential for supplying oxygen and nutrients that support regeneration [3]. By contrast, chronic wounds are characterized by endothelial dysfunction, encompassing defective progenitor cell mobilization, impaired migratory and proliferative capacity, and failure to form functional tubular networks, ultimately compromising tissue reperfusion [12]. Interestingly, initial treatment strategies that mostly focused on delivery of recombinant growth factors such as VEGF, bFGF, HIF-1α, among others, have shown limited efficacy [45]. Consequently, the development of pro-angiogenic therapeutics is of paramount clinical interest. Several studies have demonstrated that AMPs, in addition to antimicrobial properties, also have pro-healing activity, including pro-angiogenic activity, re-epithelialization, collagen deposition and tissue formation, and immunomodulatory properties, making them potential candidate for chronic wound therapy [29,30,46]. For instance, many AMPs, including hBDs, Tet213-CN, LL-37, AW1, AMP-IBP5, and Thrombocidin-1-derived peptide TC19, have been reported to promote wound healing via modulating immune response and exerting antimicrobial activities [47]. Furthermore, various AMPs, including hBDs, Histatin 1 and 2, LL-37, AMP-IBP5, LL-37, S100A7 and S100A15, synthetic HNP-1, Brevinin-2PN, PM-7, TP2-5 and TP2-6 contribute to collagen synthesis and tissue remodeling. Notably, several AMPs, such as hBDs and AMP-IBP5, exhibit pro-angiogenic activity alongside their antimicrobial and immunomodulatory functions, as well as their role in collagen synthesis and tissue regeneration [30,46].

Here we clearly show that the phosvitin-derived 29-amino-acid peptide Pt5-1c possesses strong pro-angiogenic effects, adding a new member to the list of AMPs capable of stimulating angiogenesis. The evidence supporting this property includes enhancement of mature angiogenesis in wound sites and the restoration of the formation and growth of ISVs in zebrafish with vascular defects. Furthermore, the observed increase in expression of CD31 (an endothelial cell marker) and α-SMA (a vascular smooth muscle cell marker) confirms the presence of mature neovascularization. These findings align with previous reports that combination therapies, such as the CPO/D@P/IGF-1C hydrogel with mild heat stimulation or ODEX/HA-AMP/PRP composite hydrogel, promoted the formation of more structurally mature vasculature in diabetic mice, as evidenced by elevated α-SMA and CD31 expression [7,48]. Importantly, this mature vascular patterning contrasts with the aberrant, leaky vessels often induced by VEGF monotherapy. In vitro functional assays revealed that Pt5-1c promotes endothelial cell motility, adhesion, survival and filopodia protrusion, and induces tube formation. Moreover, Pt5-1c significantly upregulates several growth factors including *VEGFA*, *PDGFA*, *FGF2*, and *EGF* in HUVECs, which may explain its pro-angiogenic effect. Meanwhile, we found that Pt5-1c could also increase the levels of *VEGFA*, *PDGFA*, *FGF2*, and *EGF* in HaCaT or HELF cells. This may contribute to the neovascularization of endothelial cells in wounds by paracrine pathways [3]. In agreement with results above, Pt5-1c also significantly enhanced the expression of *Vegfa*, *Pdgfc*, and *Fgf2* in a murine full-thickness wound model. These pro-angiogenic properties complement our earlier findings that Pt5-1c can promote re-epithelialization and granulation tissue formation in a murine full-thickness wound model, enhance adhesion, migration, and proliferation/differentiation of fibroblasts and keratinocytes, and facilitate collagen gel contraction [33,34]. It is noteworthy that the effective concentration in vivo (10 μg/mL) was higher than that used in vitro (generally up to 1 μg/mL), a difference attributable to the complex in vivo biological environment, including tissue barriers, metabolic clearance, and dilution effects [49]. All these together indicate that Pt5-1c is a very promising candidate in therapy of wound healing.

The therapeutic potential of peptides is often limited by proteolytic degradation. Structural modification such as cyclization, fluorination, amidation, D-amino acid substitution, and the incorporation of non-canonical amino acids, are proven strategies to improve AMP stability and enzymatic resistance [46,50,51]. For Pt5-1c, the C-terminal amidation and site-specific substitution stabilize the peptide into a compact α-helical conformation, thereby conferring resistance to protease recognition and cleavage [31]. In addition, biosafety and immunogenicity are critical translational considerations for AMPs [22]. Notably, shorter synthetic innate defense regulator (IDR) peptides are reported to be non-immunogenic and do not have the potentially adverse effects associated with some natural AMPs [22]. Similarly, Pt5-1c is a short (29-amino acid, 3755 Da) synthetic peptide derived from fish phosvitin, suggesting low immunogenicity. Furthermore, Pt5-1c exhibits no hemolytic activity in vitro and induces no signs of tissue damage, fibrosis, or morphological abnormalities in major organs in vivo, indicating a favorable safety profile. Collectively, its potent pro-angiogenic and pro-healing efficacy, enhanced stability through rational design, and demonstrated biosafety profile establish Pt5-1c as a highly promising therapeutic candidate for wound healing. However, a limitation of our study is that the pro-angiogenic efficacy of Pt5-1c were not compared with the positive control (e.g., VEGF, bFGF).

**Figure 6:**
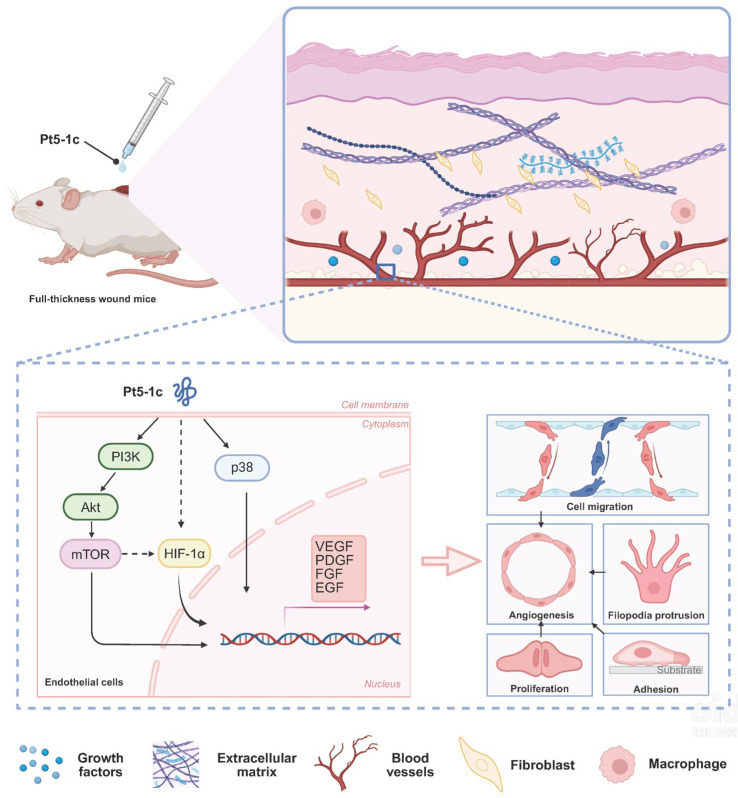
A schematic representation illustrates how Pt5-1c activates both PI3K/AKT/mTOR and p38 MAPK pathways as well as HIF-1-VEGF axis simultaneously or sequentially, which is crucial for endothelial cell proliferation, migration, and tube formation. Solid arrow represents mechanisms verified by our inhibitor/Western blot experiments, while dashed arrow represents proposed mechanisms supported by cited literature [56,57].

Pt5-1c has pro-angiogenic activity both in vitro and in vivo, promoting our investigation into its underlying mechanism to assess its broader therapeutic potential. Our results show that increased phosphorylation of AKT, mTOR, and p38 occurs within minutes of Pt5-1c treatment, and pharmacological inhibition of PI3K/AKT/mTOR and p38 MAPK pathways abrogates migratory and tube-forming responses. Previous studies found that in the wounded skin of mice, the expression of p-PI3K and p-AKT was regulated to peak during the inflammatory and proliferation phase [52]. Activating PI3K/AKT/mTOR signaling pathway is beneficial for normal and chronic wound repair as it mediates cell proliferation, migration, apoptosis, and collagen deposition during healing [52]. Similarly, p38 MAPK pathways are central to endothelial cell survival, growth, and motility [17,53], and has been reported to play a vital role in postischemic neuroprotection and neurodisorder tolerance phenomena [54]. Moreover, molecular docking reveals potential binding interactions between Pt5-1c and PI3K, AKT, mTOR, and p38, offering a structural basis for its pro-angiogenic activity. These suggest that Pt5-1c promotes endothelial cell motility, growth and survival via activation of key signaling cascades including PI3K/AKT/mTOR and p38 MAPK pathways, although whether it affects the half-life of these kinases remains to be determined. Another interesting finding of our study is that Pt5-1c upregulates expression of *HIF-1α*, a master transcriptional regulator of angiogenesis. It has been documented that HIF-1 can enhance VEGF transcription via binding to hypoxia-responsive elements, and HIF-1-mediated VEGF signaling coordinates vascularization with other regenerative processes such as neurogenesis [55]. It is thus also possible that Pt5-1c can initiate angiogenesis through HIF-1-VEGF axis activation, though further study is still needed. Emerging evidence highlights a complex crosstalk between the mTOR and HIF-1α pathways, forming a self-reinforcing positive feedback loop [56]. Therefore, mechanistically we propose that Pt5-1c activates both PI3K/AKT/mTOR and p38 MAPK pathways as well as the HIF-1-VEGF axis simultaneously or sequentially, which in turn collectively stimulates a concerted generation of major pro-angiogenic factors including VEGF, PDGF, FGF and EGF that then trigger cell migration, adhesion, proliferation and growth, eventually enhancing angiogenesis (Figure 6). However, whether Pt5-1c initiates these signals by binding to a specific receptor remains an important question for further research.

## 5. Conclusions

In conclusion, this study highlights Pt5-1c as a novel candidate of pro-angiogenic agents for vascular regenerative therapy. Its ability to simultaneously promote multiple aspects of endothelial cell function and upregulate numerous pro-angiogenic growth factors mirrors the pleiotropy of other AMPs such as tylotoin, histatin-1, hBD-3, and AMP-IBP5 [42,58,59,60], yet through a distinct and complementary mechanism. Future work may employ integrated multi-omics and functional genetics approaches to precisely delineate the molecular targets and signaling hierarchy of Pt5-1c.

## Figures and Tables

**Table 1:** Primer sequences for qRT-PCR in human.

Gene	Forward (5′-3′)	Reverse (5′-3′)
*VEGF-A*	GAGGGCAGAATCATCACGAA	GGTCTCGATTGGATGGCAGTA
*PDGF-A*	GACGGTCATTTACGAGATTCCT	CTCCTCTAACCTCACCTGGACT
*FGF-2*	GAGCGACCCTCACATCAA	CGTTTCAGTGCCACATACC
*EGF*	TGGATGGTTCAAAACGCCGAAG	ACGTACTCTATCTTTGCCAGTCCT
*HIF-1α*	CTCGGCGAAGTAAAGAAT	ATCCAAATCACCAGCATC
*GAPDH*	GGAGTCCACTGGCGTCTT	GAGTCCTTCCACGATACCAA

**Table 2:** Primer sequences for qRT-PCR in mouse.

Gene	Forward (5′-3′)	Reverse (5′-3’)
*Vegfa*	GTGCACTGGACCCTGGCTTTA	GGTCTCAATCGGACGGCAGTA
*Pdgfc*	GTGGAGGAAATTGTGCCTGT	TCCAGAGCCACATCAGTGAG
*Fgf2*	GGACGGCTGCTGGCTTCTAA	CCAGTTCGTTTCAGTGCCACATAC
*Gapdh*	TCCCAGCTTAGGTTCATCAGGTAAA	CAATCTCCACTTTGCCACT

## Data Availability

The original contributions presented in this study are included in the article/Supplementary Materials. Further inquiries can be directed to the corresponding authors.

## Supplementary Materials

The following supporting information can be downloaded at: https://www.mdpi.com/article/10.3390/biom16010065/s1, Figure S1: Representative images and quantitative analysis of immunohistochemistry staining of CD31 and α-SMA of wound sections on D12 post-injury. Scale bar: 50 μm; Figure S2: Western blotting was used to analyze the protein expression level of AKT after HUVECs were treated with 1μg/ml Pt5-1c for a certain period of time. Figure S3: The structure of Pt5-1c. And Western blot original images.
