# Supplementary material for: Phosvitin-Derived Peptide Pt5-1c Is a Pro-Angiogenic Agent Capable of Enhancing Wound Healing
Source: Biomolecules. 2025 Dec 31;16(1):65. doi: 10.3390/biom16010065 (PMC12838811; doi:10.3390/biom16010065)
Supplement: Supplementary file 1 [file biomolecules-16-00065-s001.zip › biomolecules-4021671-supplementary/biomolecules-4021671-Original Images for Blots.pdf]

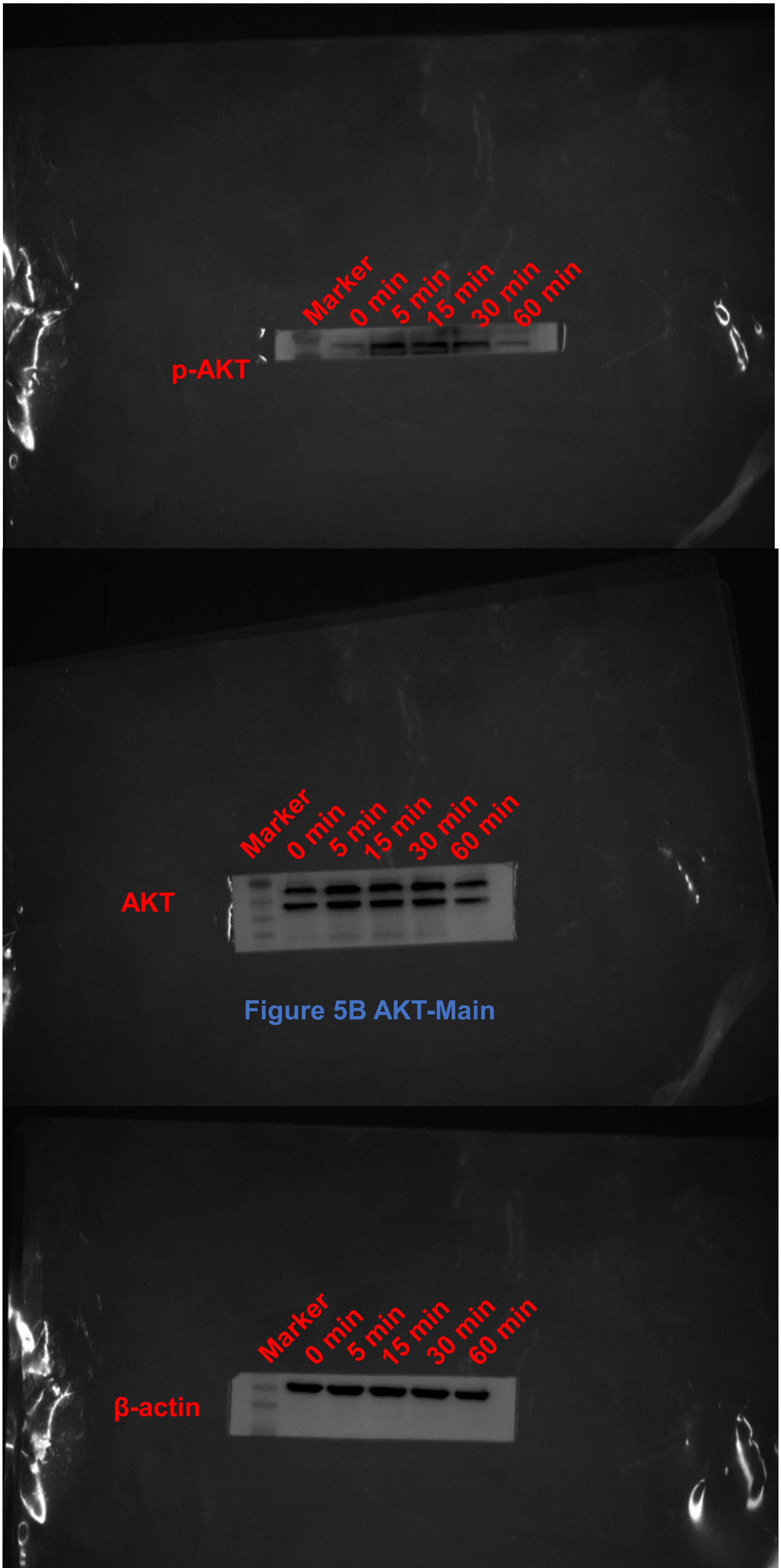

These original images correspond to Figure 5B in the main article.

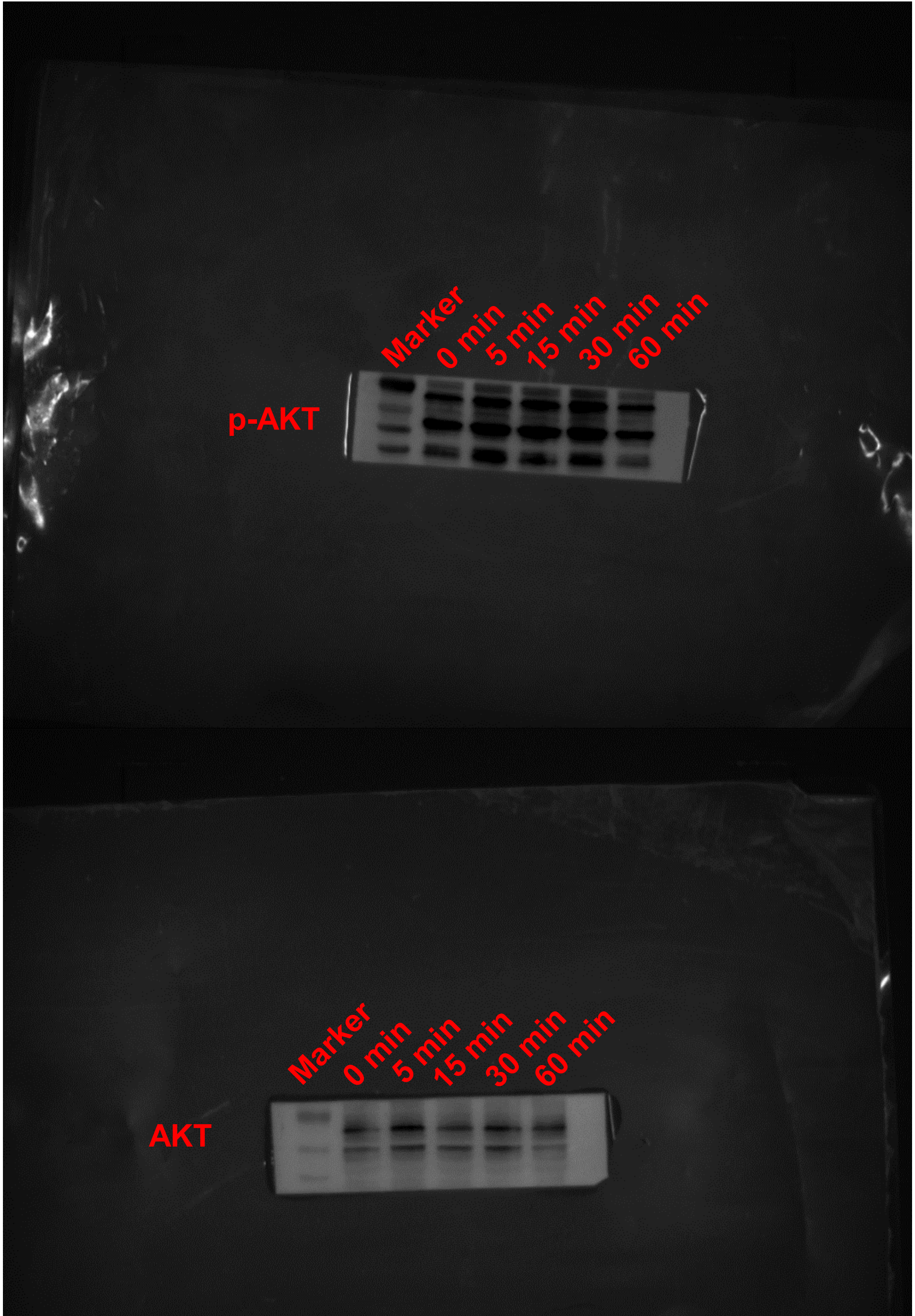

These original images correspond to Figure 5B in the main article.

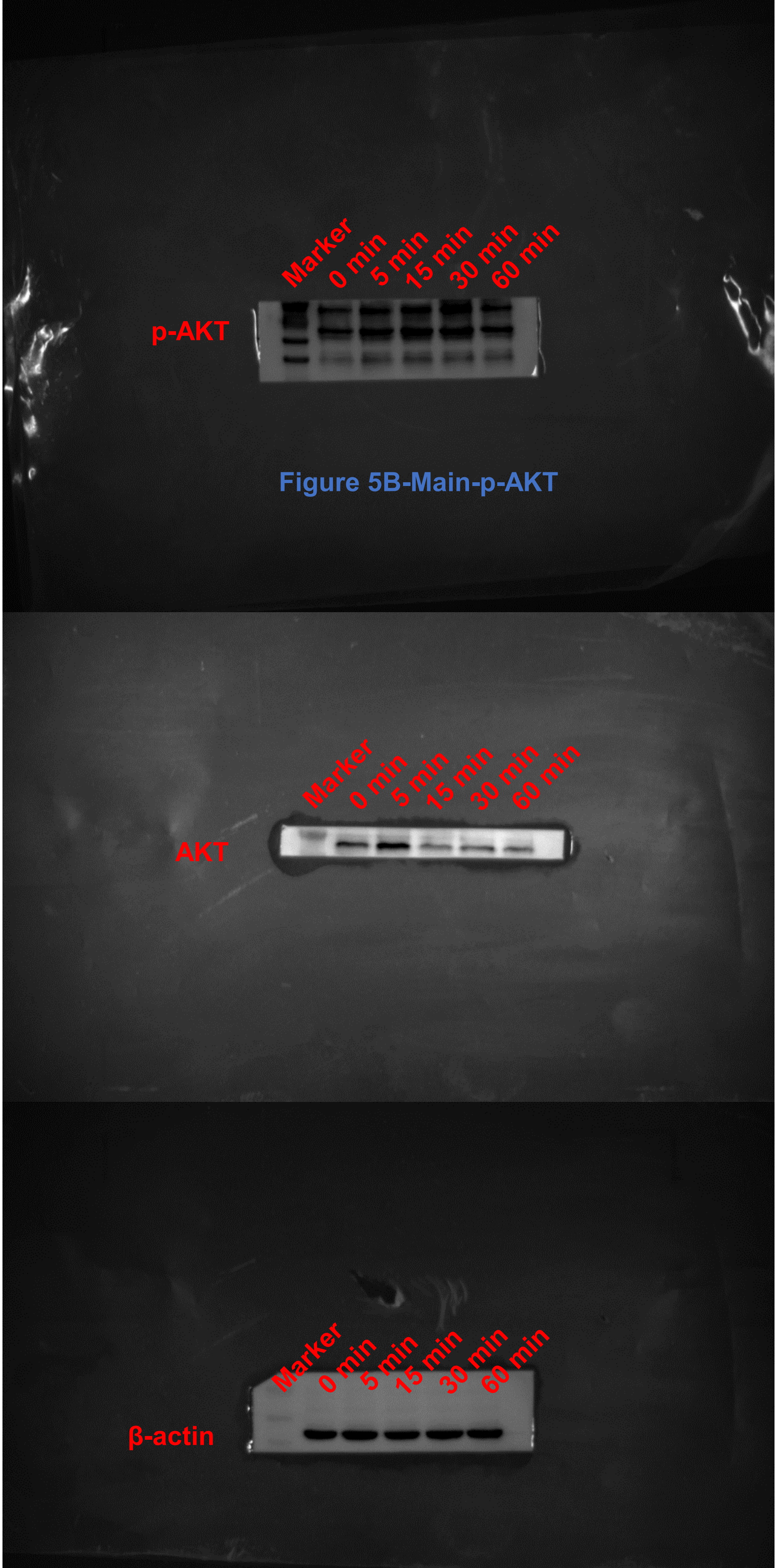

These original images correspond to Figure 5B in the main article.

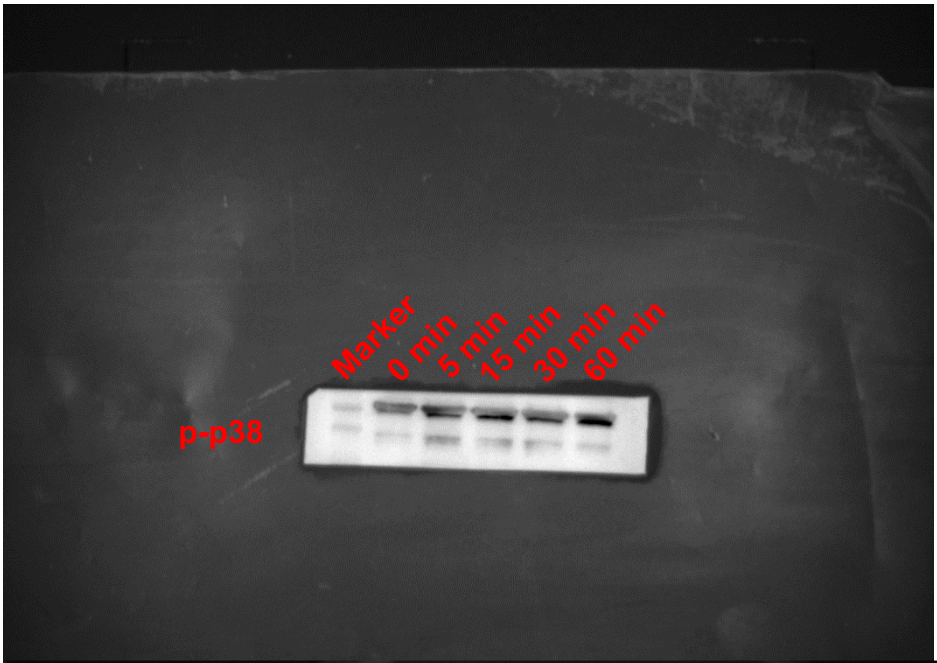

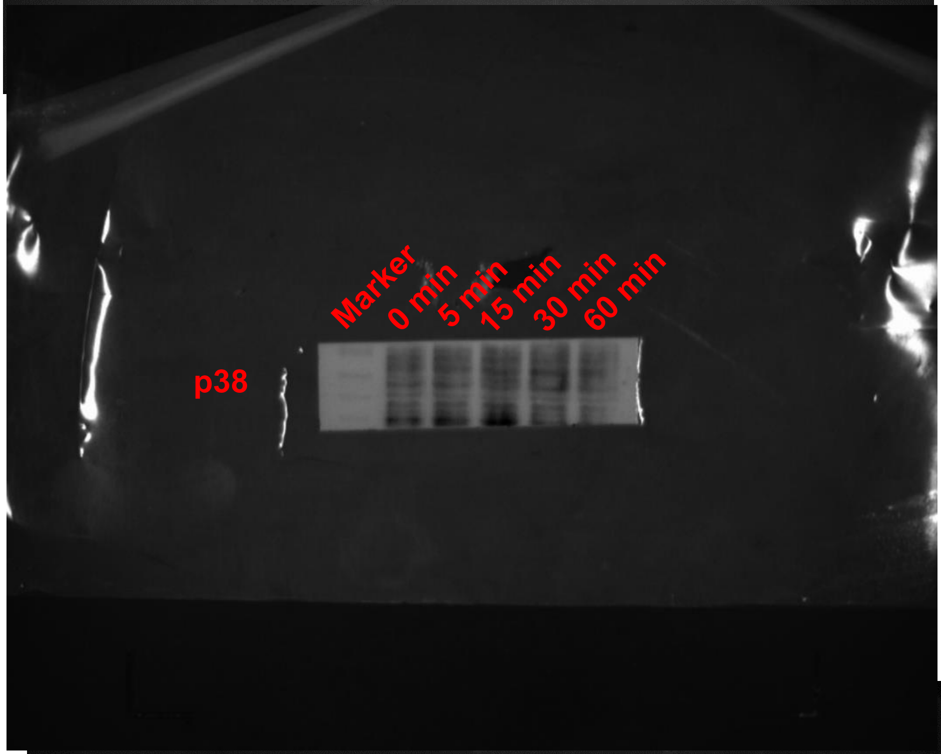

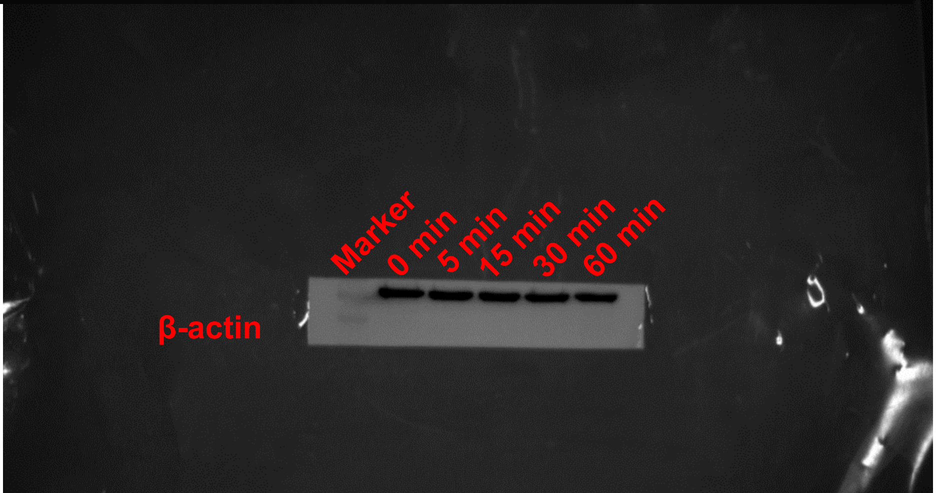

These original images correspond to figure 5B in the main article.

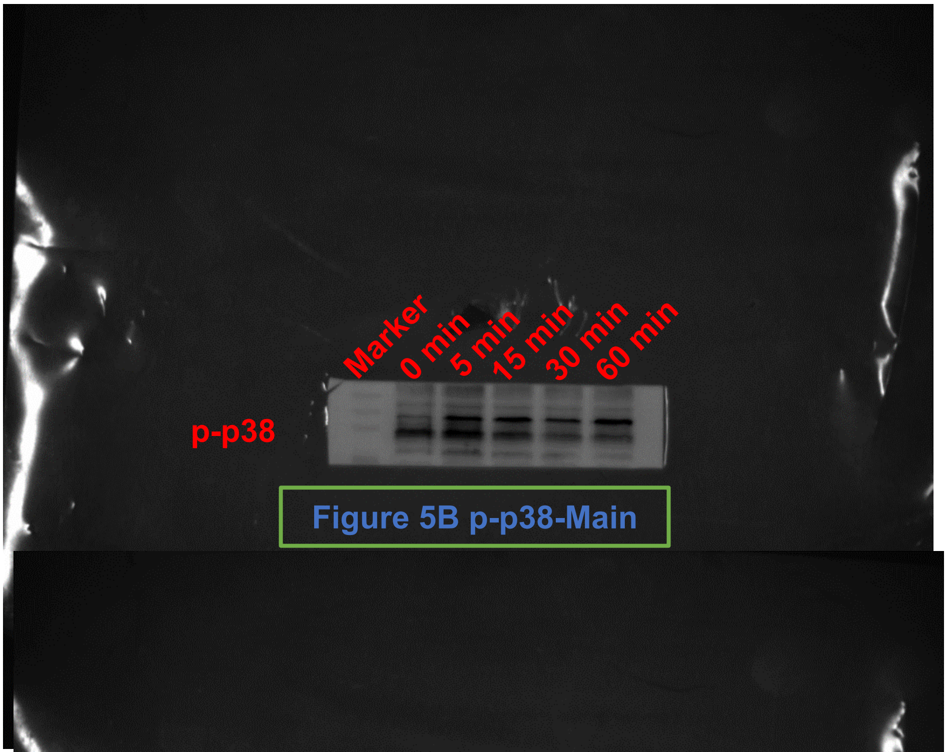

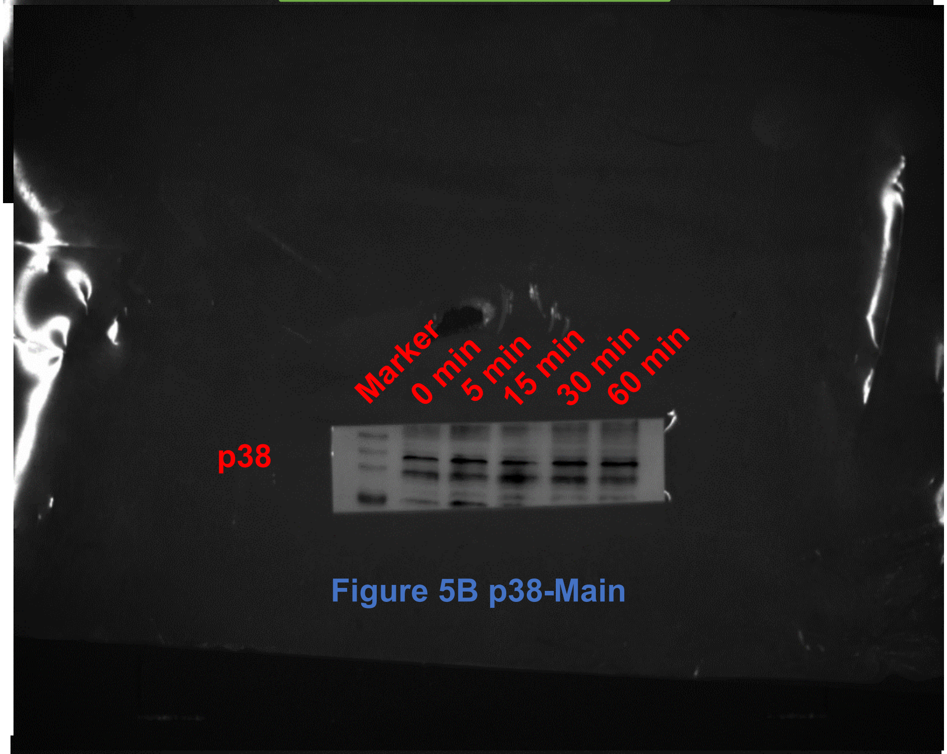

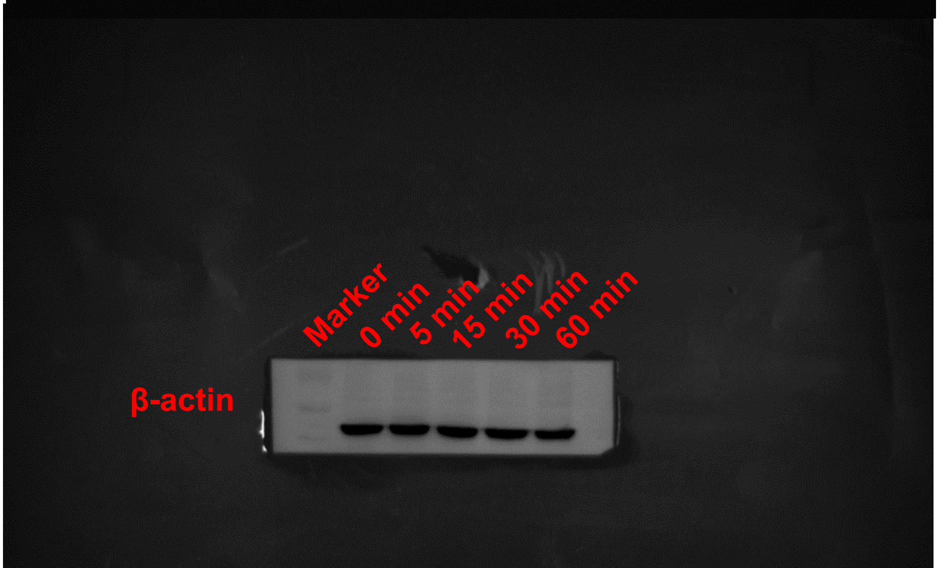

These original images correspond to Figure 5B in the main article.

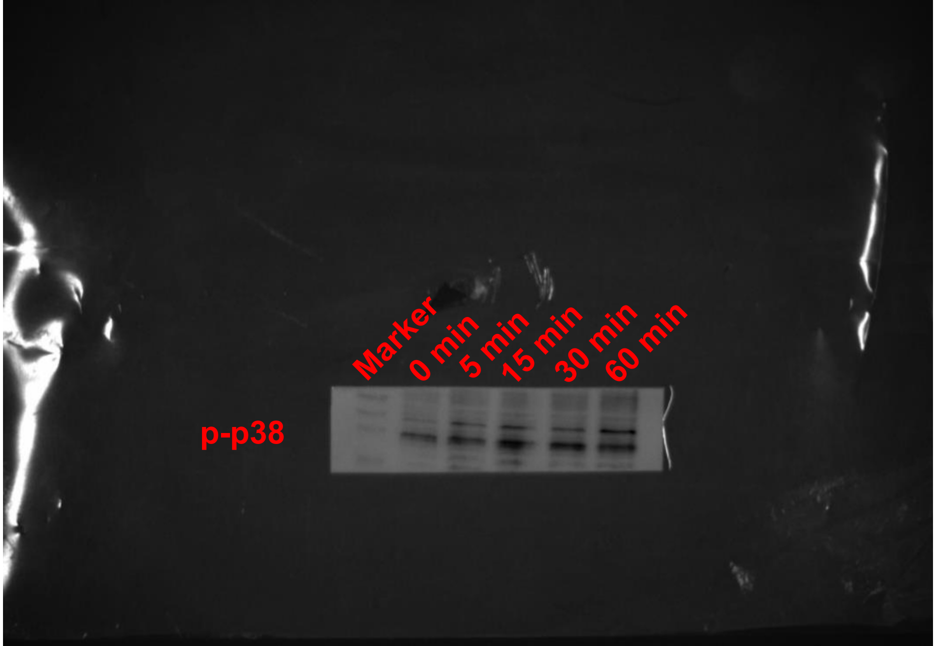

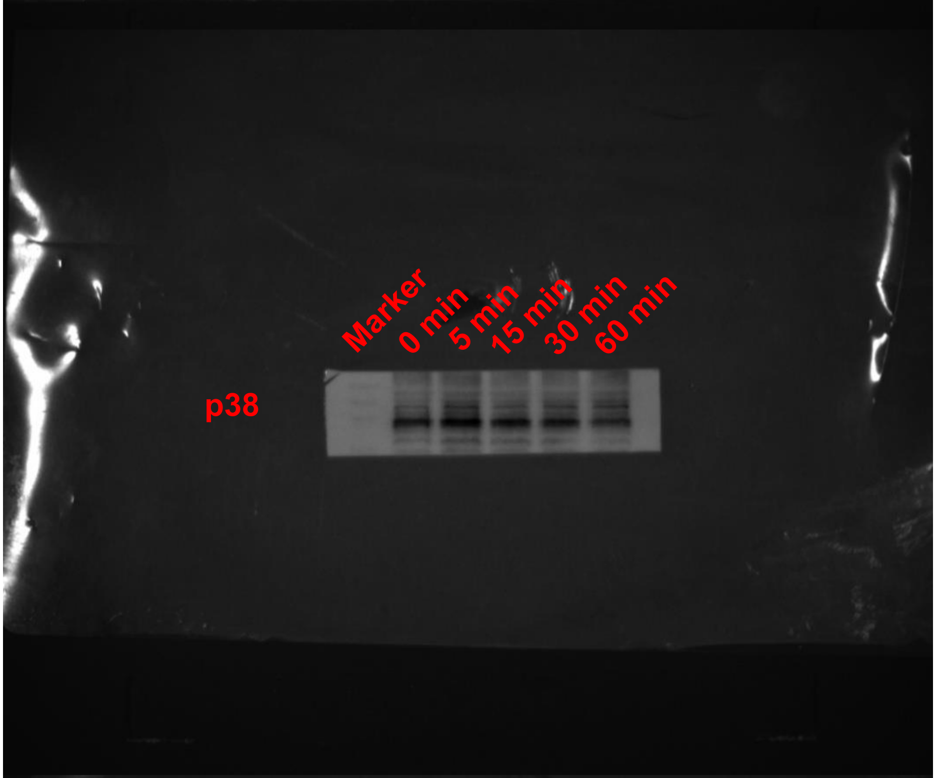

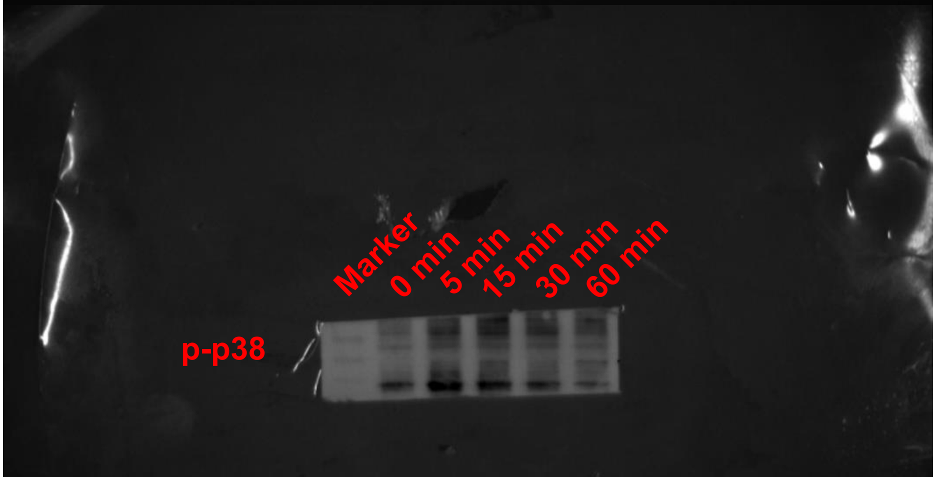

These original images correspond to Figure 5B in the main article.

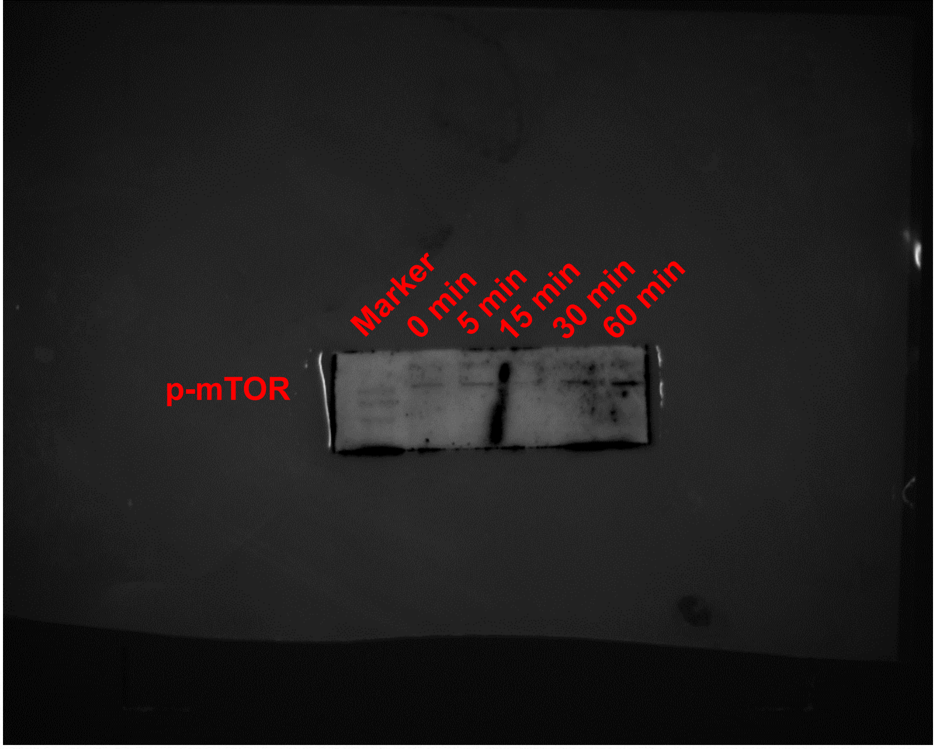

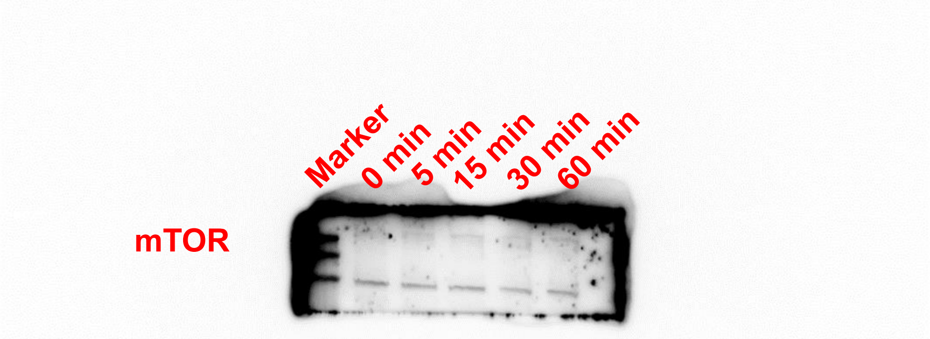

Figure 5B mTOR-Main

The mTOR blot bands obtained with a short exposure time.

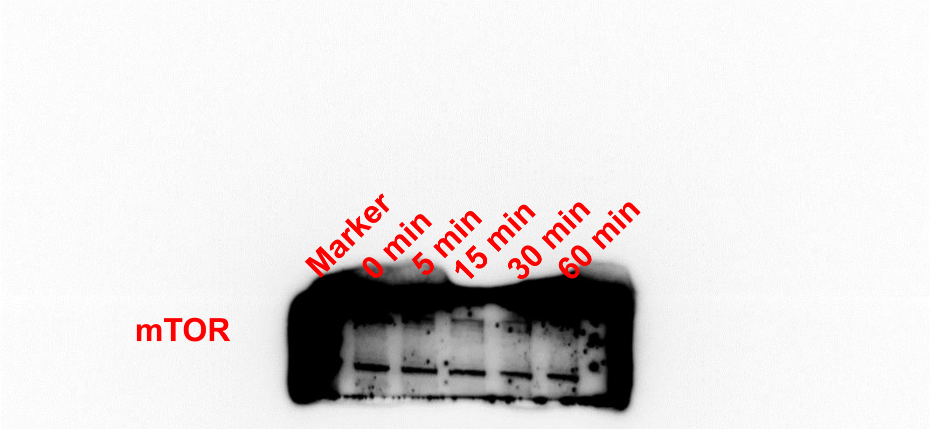

Figure 5B mTOR-Main

The mTOR blot bands obtained with a long exposure time.

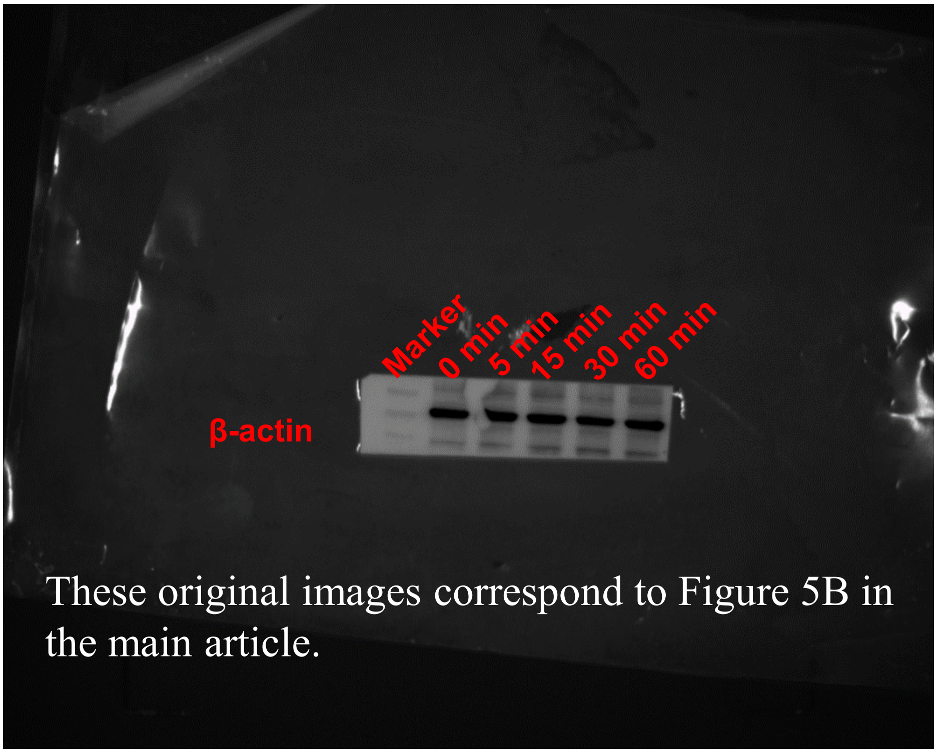

These original images correspond to Figure 5B in the main article.

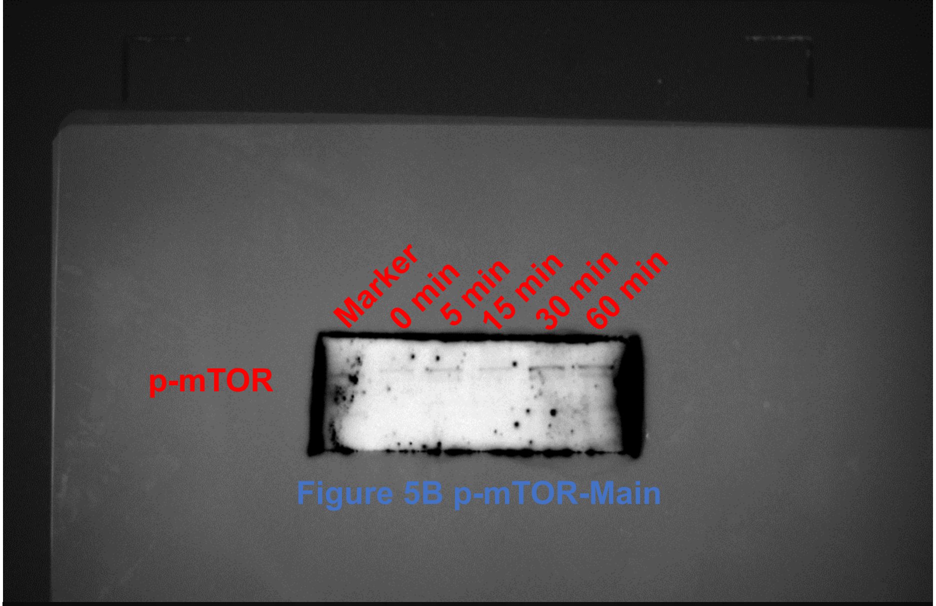

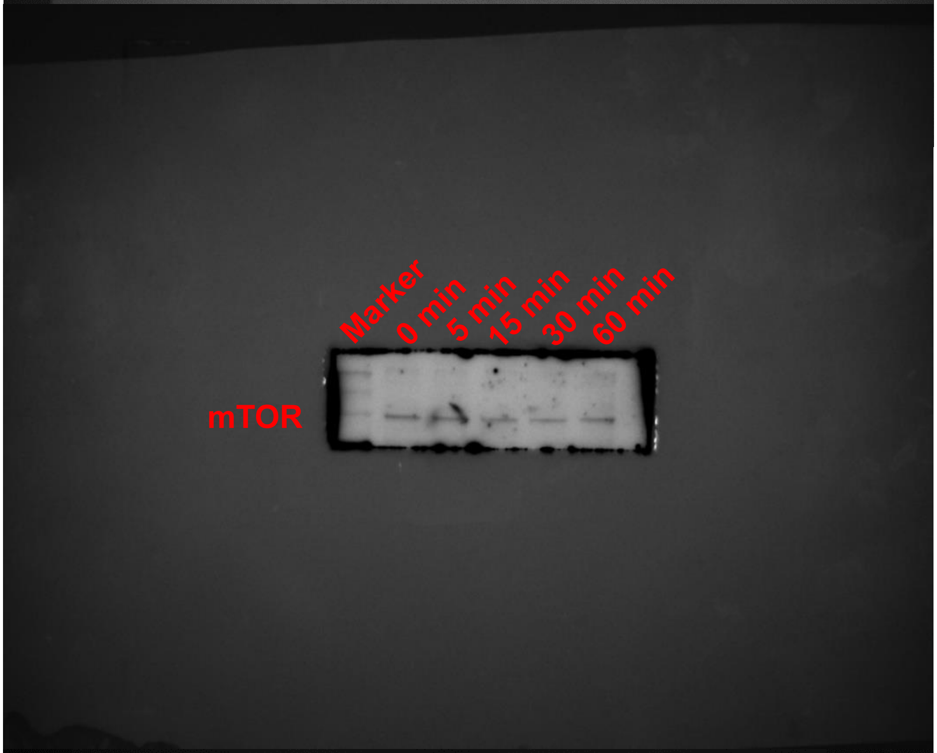

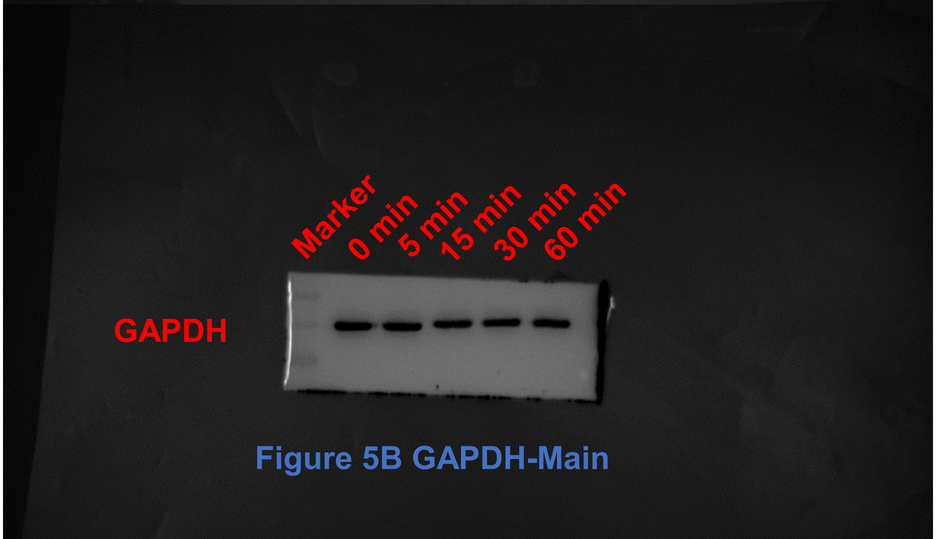

These original images correspond to Figure 5B in the main article.

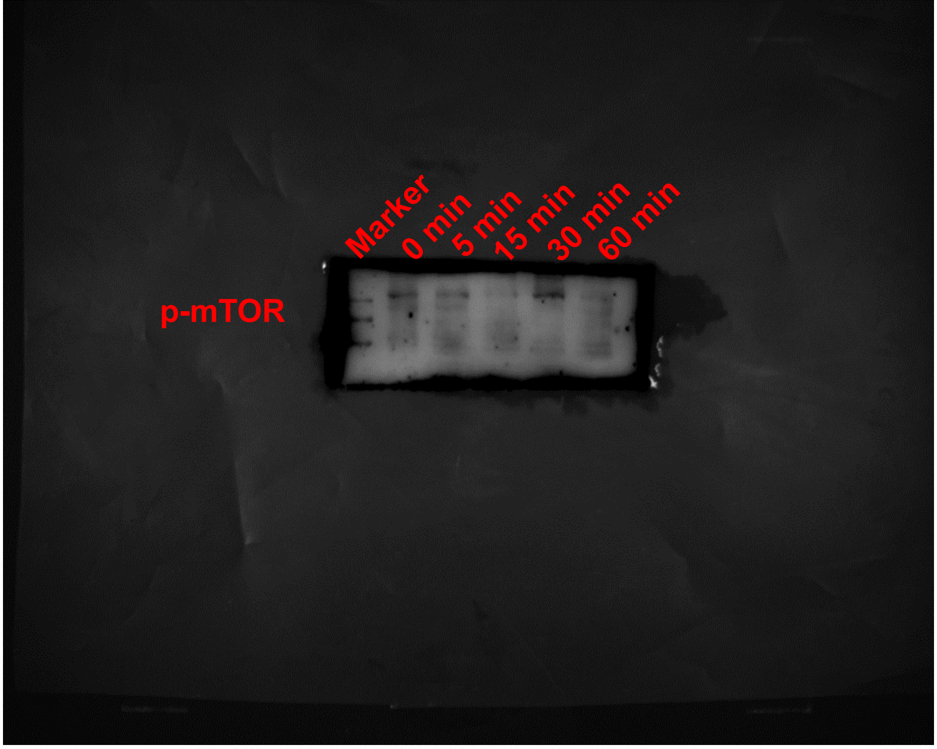

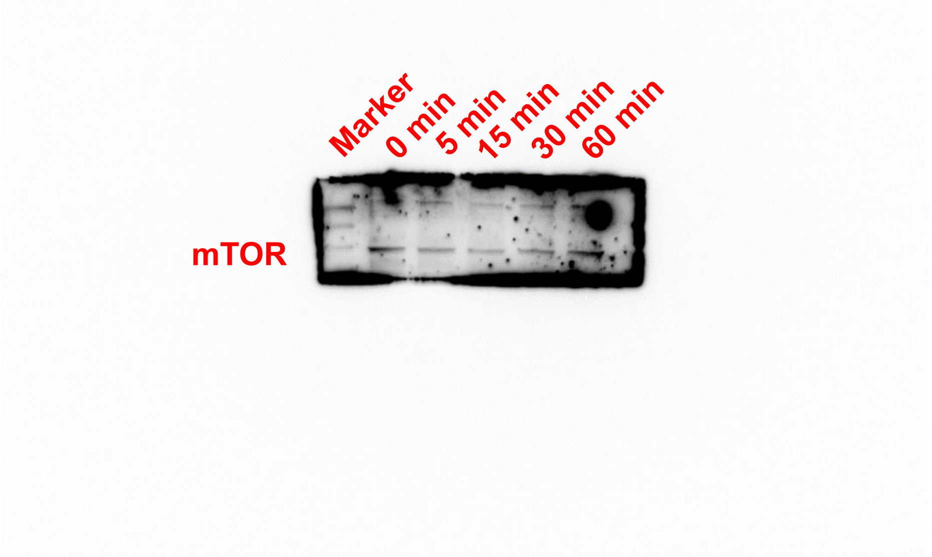

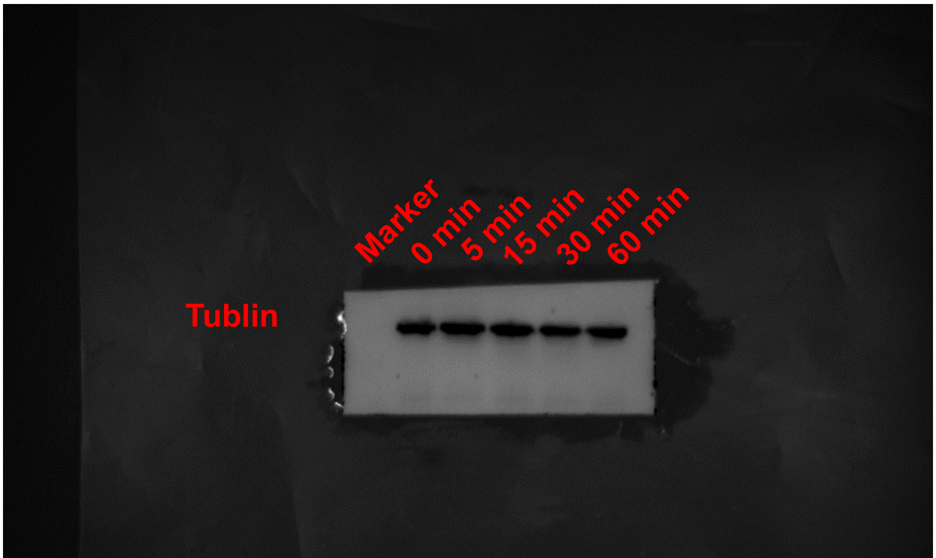

These original images correspond to Figure 5B in the main article.

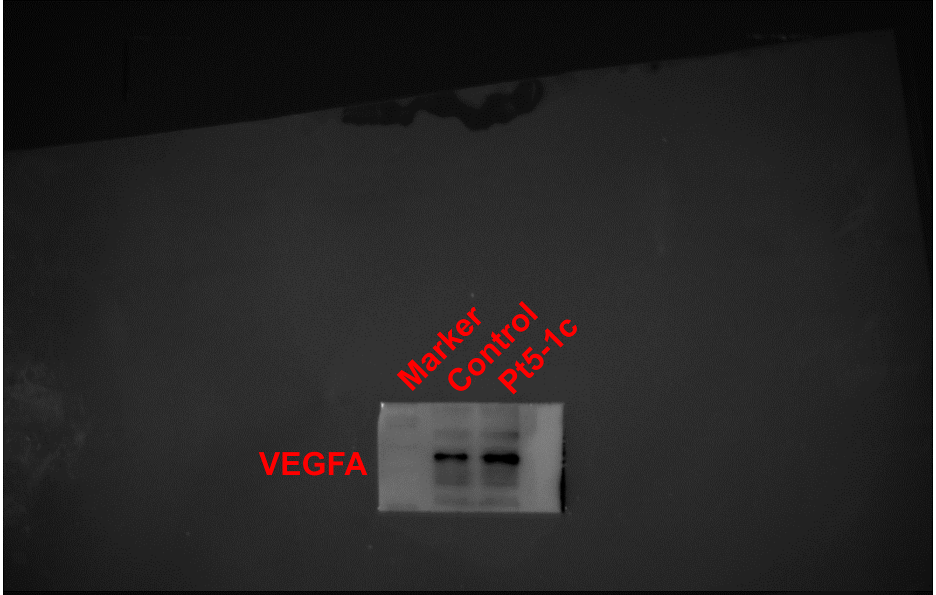

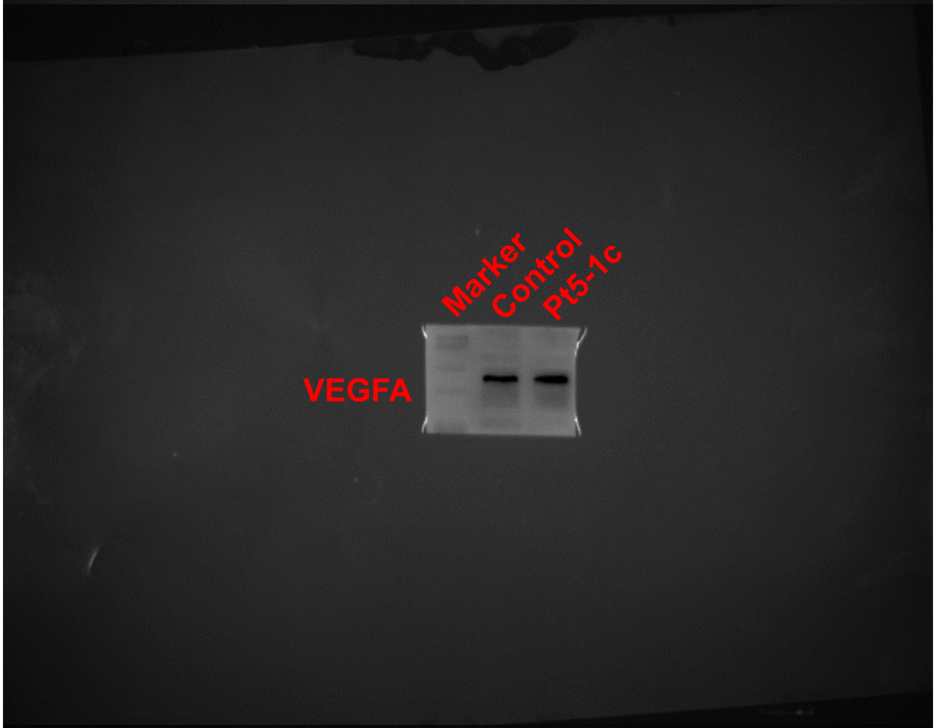

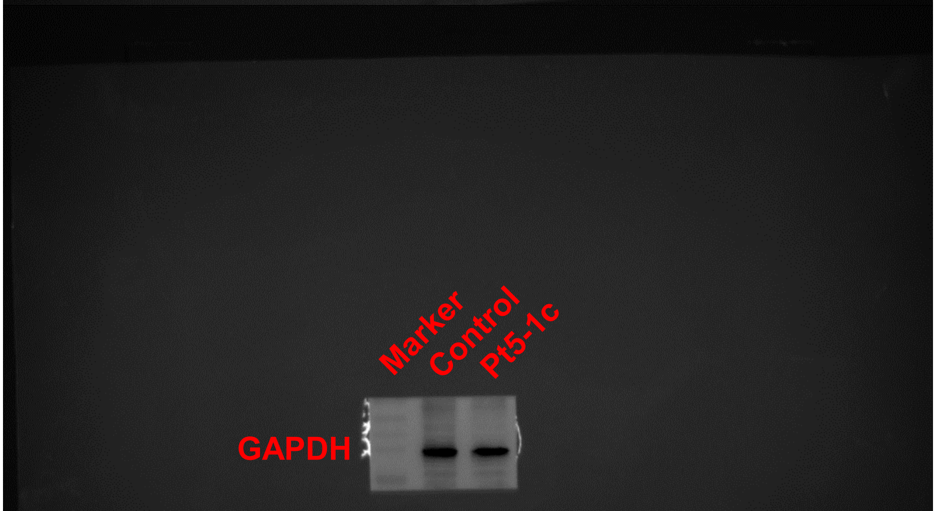

Figure 2N GAPDH-Main

These original images correspond to figure 2N in the main article.

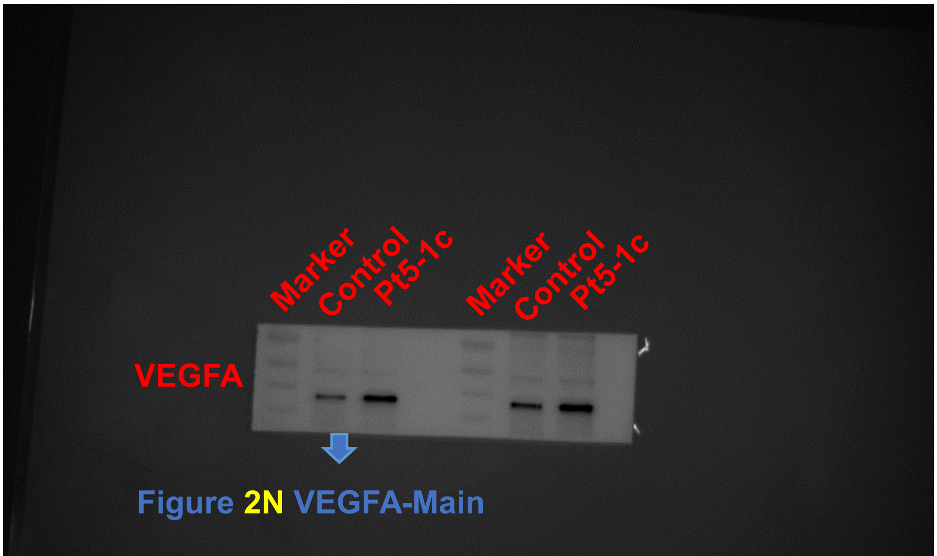

These original images correspond to figure 2N in the main article.

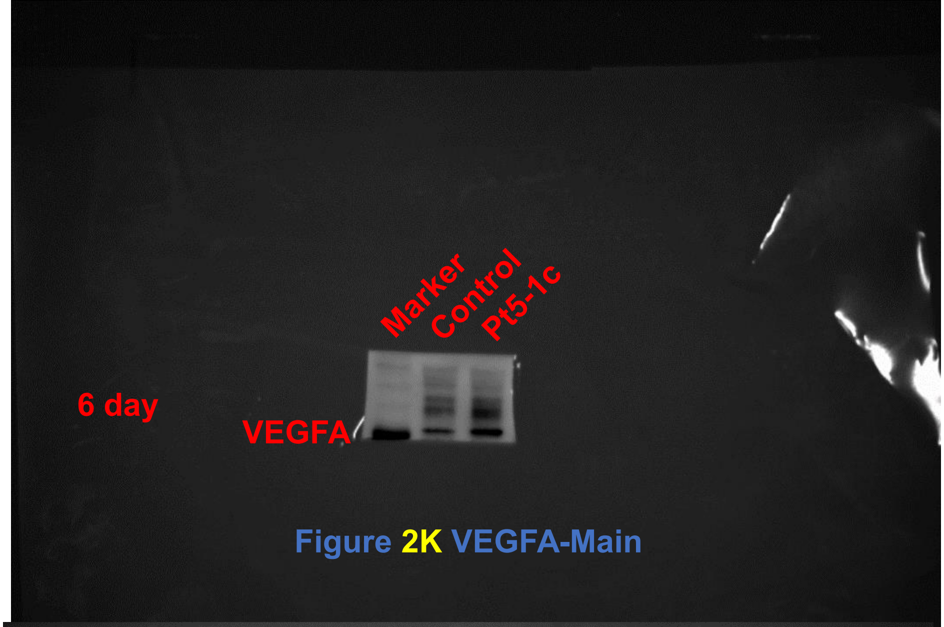

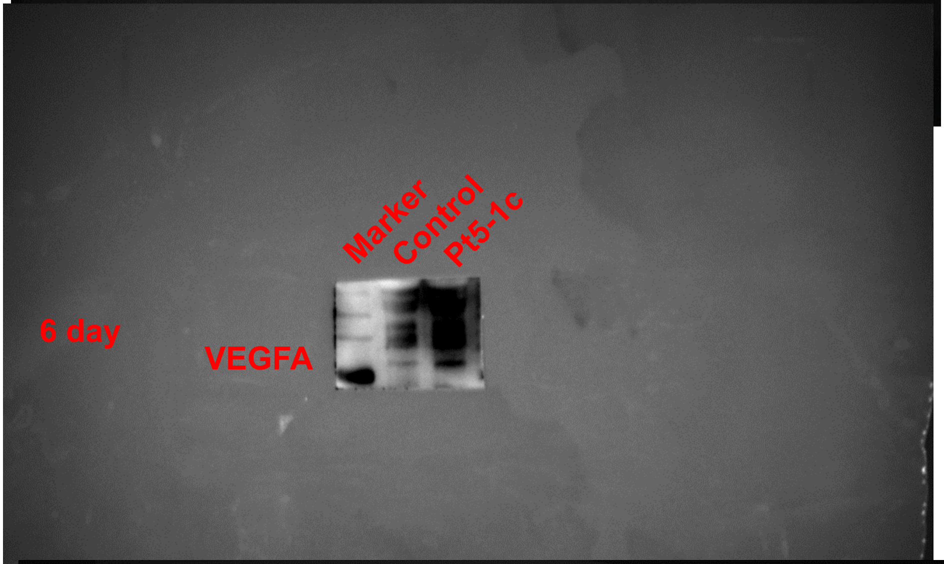

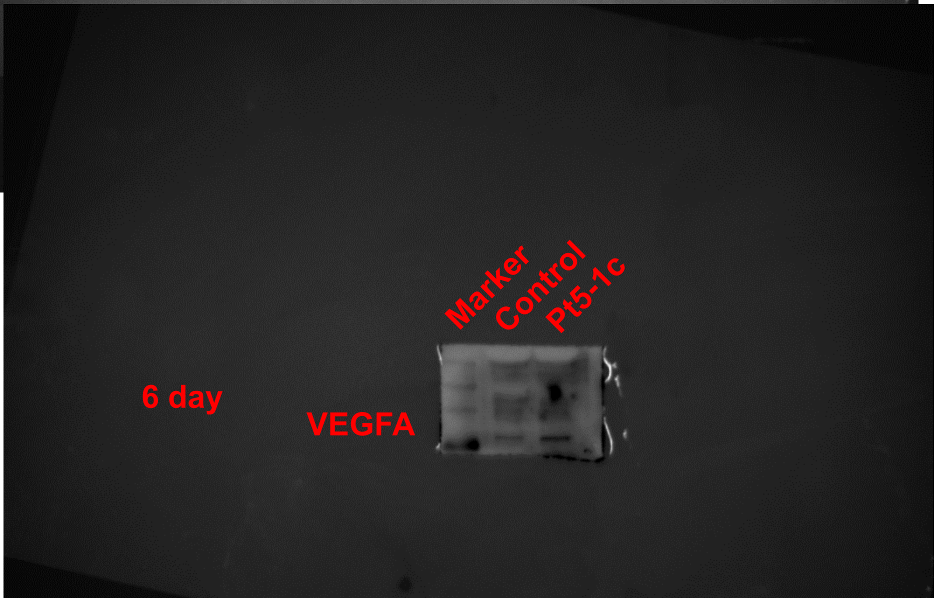

These original images correspond to figure 2K in the main article.

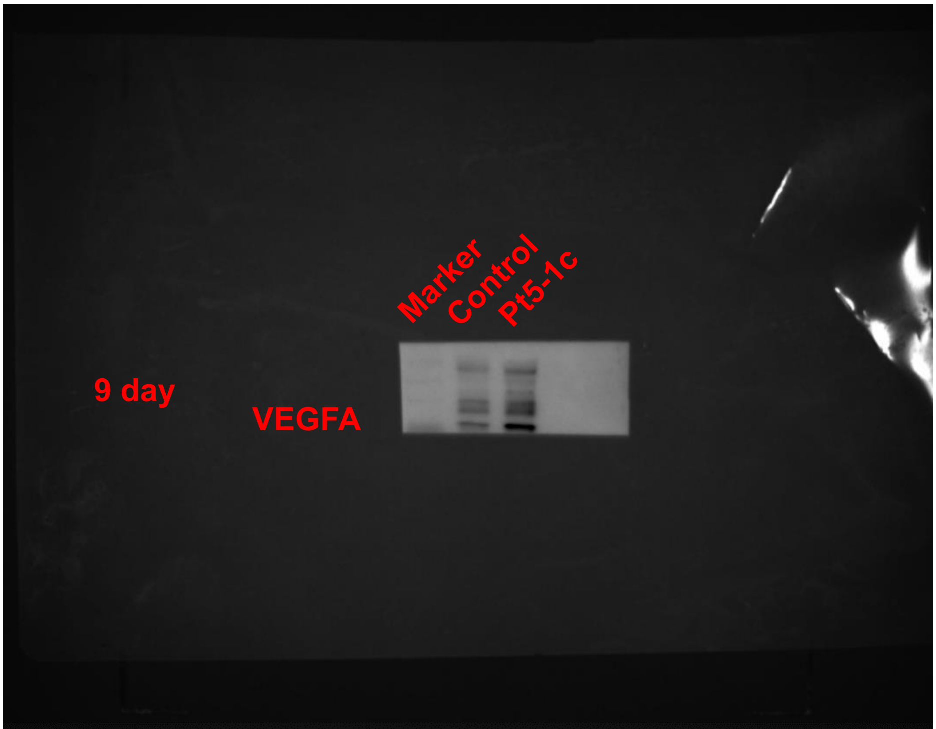

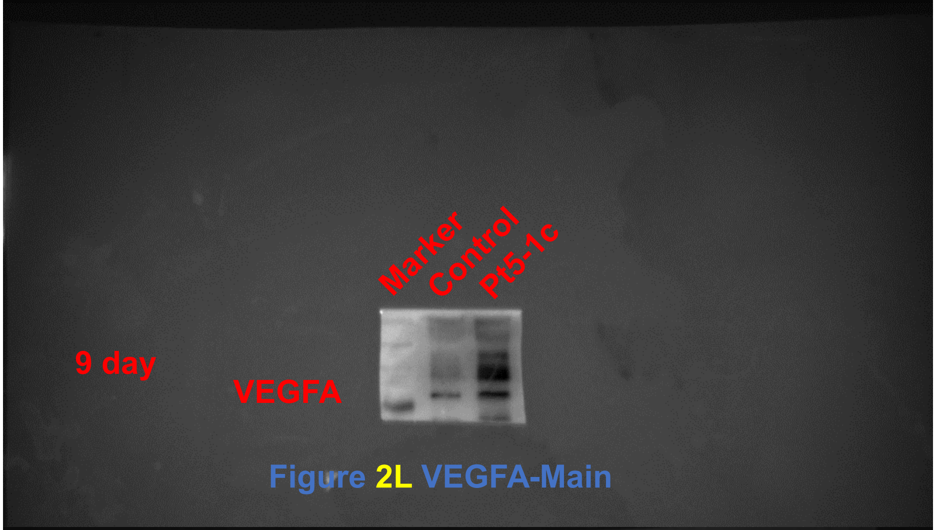

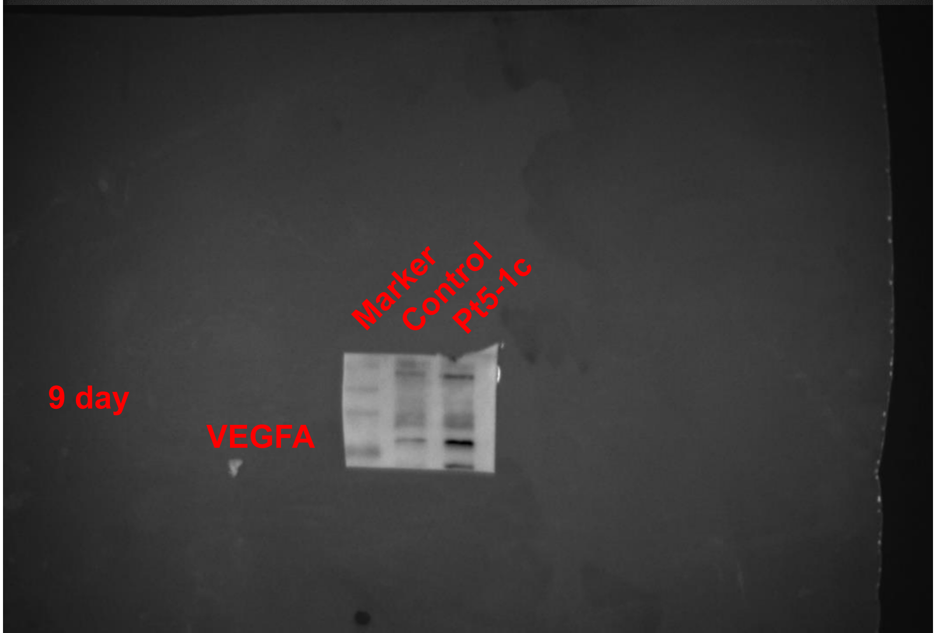

These original images correspond to figure 2L in the main article.

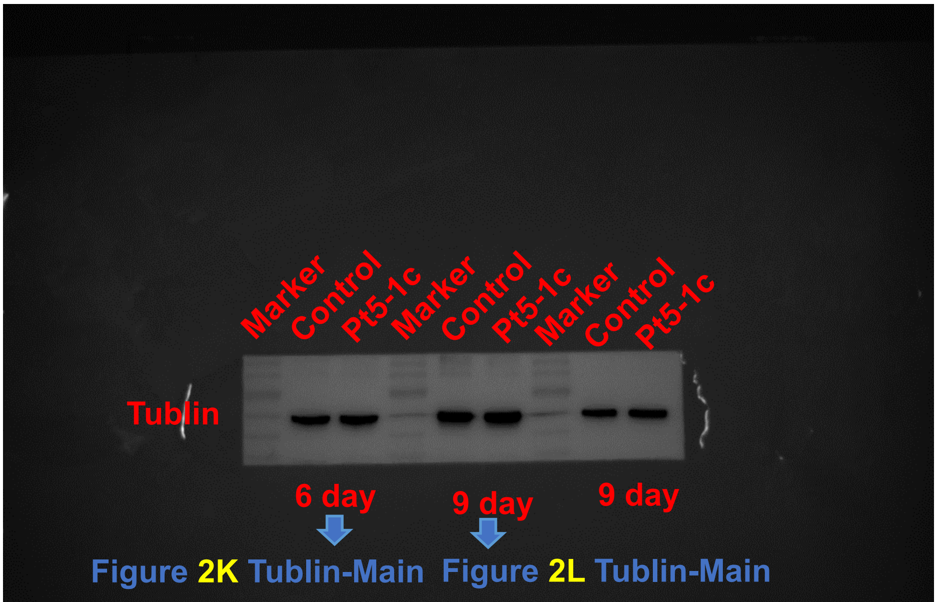

These original images correspond to figure 2K,L in the main article.
