# Supplementary material for: Phosvitin-Derived Peptide Pt5-1c Is a Pro-Angiogenic Agent Capable of Enhancing Wound Healing
Source: Biomolecules. 2025 Dec 31;16(1):65. doi: 10.3390/biom16010065 (PMC12838811; doi:10.3390/biom16010065)
Supplement: Supplementary file 1 [file biomolecules-16-00065-s001.zip › biomolecules-4021671-supplementary/biomolecules-4021671-supplementary.pdf]

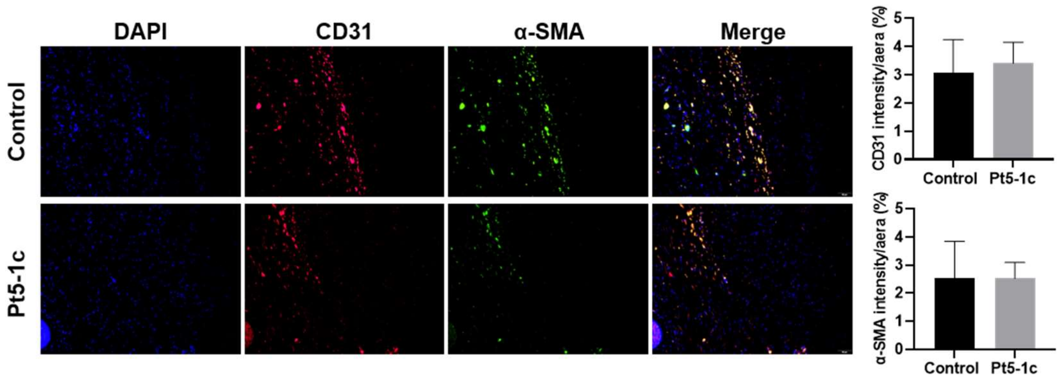

Figure S1. Representative images and quantitative analysis of immunohistochemistry staining of CD31 and α-SMA of wound sections on D12 post-injury. Scale bar: 50 μm.

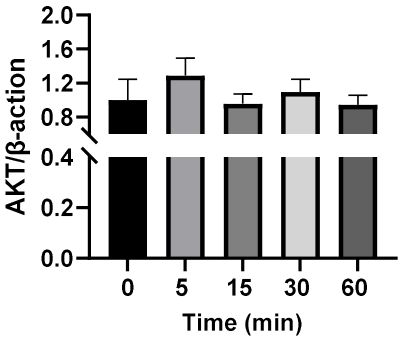

Figure S2. Western blotting was used to analyze the protein expression level of AKT after HUVECs were treated with 1μg/ml Pt5-1c for a certain period of time.

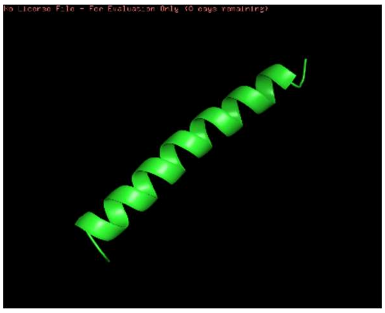

Figure S3. The structure of Pt5-1c.
